# AUTS2 Governs Cerebellar Development, Purkinje Cell Maturation, Motor Function and Social Communication

**DOI:** 10.1016/j.isci.2020.101820

**Published:** 2020-11-18

**Authors:** Kunihiko Yamashiro, Kei Hori, Esther S.K. Lai, Ryo Aoki, Kazumi Shimaoka, Nariko Arimura, Saki F. Egusa, Asami Sakamoto, Manabu Abe, Kenji Sakimura, Takaki Watanabe, Naofumi Uesaka, Masanobu Kano, Mikio Hoshino

**Affiliations:** 1 Department of Biochemistry and Cellular Biology, National Institute of Neuroscience, National Center of Neurology and Psychiatry (NCNP), Tokyo 187-8502, Japan; 2 Department of NCNP Brain Physiology and Pathology, Graduate School of Medical and Dental Sciences, Tokyo Medical and Dental University, Tokyo 113-8510, Japan; 3 Brain Mechanism for Behavior Unit, Okinawa Institute of Science and Technology Graduate University, Okinawa 904-0495, Japan; 4 Department of Neurophysiology, Graduate School of Medicine, The University of Tokyo, Tokyo 113-0033, Japan; 5 Department of Animal Model Development, Brain Research Institute, Niigata University, Niigata 951-8585, Japan; 6 Department of Cognitive Neurobiology, Graduate School of Medical and Dental Sciences, Tokyo Medical and Dental University, Tokyo 113-8510, Japan

**Keywords:** Molecular Neuroscience, Developmental Neuroscience, Cellular Neuroscience

## Abstract

*Autism susceptibility candidate 2* (*AUTS2*), a risk gene for autism spectrum disorders (ASDs), is implicated in telencephalon development. Because AUTS2 is also expressed in the cerebellum where defects have been linked to ASDs, we investigated AUTS2 functions in the cerebellum. AUTS2 is specifically localized in Purkinje cells (PCs) and Golgi cells during postnatal development. *Auts2* conditional knockout (cKO) mice exhibited smaller and deformed cerebella containing immature-shaped PCs with reduced expression of *Cacna1a*. *Auts2* cKO and knock-down experiments implicated AUTS2 participation in elimination and translocation of climbing fiber synapses and restriction of parallel fiber synapse numbers. *Auts2* cKO mice exhibited behavioral impairments in motor learning and vocal communications. Because *Cacna1a* is known to regulate synapse development in PCs, it suggests that AUTS2 is required for PC maturation to elicit normal development of PC synapses and thus the impairment of *AUTS2* may cause cerebellar dysfunction related to psychiatric illnesses such as ASDs.

## Highlights

• Loss of *Auts2* leads to the reduction of cerebellar size
• AUTS2 promotes the dendritic maturation of Purkinje cells
• AUTS2 participates in PF and CF synapse development of Purkinje cells
• *Auts2* cKO mice exhibit the impaired motor learning and vocal communications

## Graphical Abstract

**Figure:**
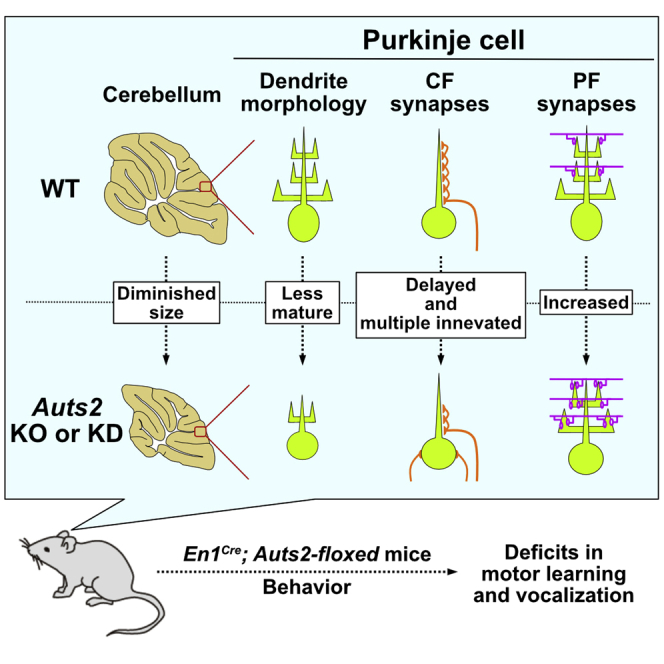

## Introduction

The cerebellum is a well-defined brain region known to control motor coordination and function. The cerebellar cortex consists of a uniform three-layered structure: the molecular layer (ML), Purkinje cell layer (PCL), and granule cell layer (GCL) (Ito, 2006). Because its highly stereotyped cytoarchitecture is composed of fewer types of neuronal cells compared with other brain regions, the cerebellum has been used as a good model system to study neurogenesis and cell morphogenesis as well as circuit assembly (Sillitoe and Joyner, 2007). Among neurons in the cerebellar cortex, Purkinje cells (PCs) are the sole output neurons that extend a long axon to deep cerebellar nuclei (DCN) neurons (White and Sillitoe, 2013). In mouse brains, PCs are generated at the ventricular zone of the cerebellar primordia during embryonic (E) 11–13 days and then migrate and differentiate until birth (Altman and Bayer, 1978; Yuasa et al., 1991). During the first three weeks of postnatal development, PCs form apical stem dendrites with extremely elaborated branches. Each PC receives excitatory presynaptic inputs from a single climbing fiber (CF) originating from a neuron in the inferior olivary nucleus (ION) and simultaneously accepts inputs from the multiple parallel fibers (PFs) projecting from granule cells (GCs). Accumulating evidence demonstrates that the cerebellum is increasingly appreciated as a potential regulator for high-order brain functions. Functional magnetic resonance imaging (fMRI) studies on human subjects have revealed that the activation of the cerebellum is associated with social cognition and emotional processing (Schmahmann and Caplan, 2006; Van Overwalle et al., 2014). Accordingly, isolated cerebellar injury or cerebellar lesions have been linked to various types of cognitive and social impairments (Limperopoulos et al., 2007; Schmahmann and Sherman, 1998). Postmortem studies in individuals with autism spectrum disorders (ASDs) displayed cerebellar PC loss (Amaral et al., 2008; Bauman and Kemper, 2005). In addition, animal models of various neurological disorders revealed that a reduction in the number or dysfunction of PCs leads to abnormal social behaviors (Tsai et al., 2012). However, despite the significance of proper development and function of PCs for socio-cognitive processes in the cerebellum, the pathological mechanisms underlying how impairments of development or function of PCs contribute to neurological disorders remain to be clarified.

*Autism susceptibility candidate 2* (*AUTS2*) (also termed “activator of transcription and developmental regulator”) has been identified in human genetic studies as a risk gene for numerous types of psychiatric illnesses, including ASDs, intellectual disabilities (IDs), and schizophrenia (Hori and Hoshino, 2017; Oksenberg and Ahituv, 2013). In addition, the genomic structural variants in the *AUTS2* locus have been associated with multiple types of neurological disorders such as attention deficit hyperactivity disorder (ADHD) and dyslexia (Elia et al., 2010; Girirajan et al., 2011). Moreover, *AUTS2* has been implicated in other neuropathological conditions such as epilepsy, motor delay, and language delay (Mefford et al., 2010; Sengun et al., 2016; Talkowski et al., 2012). *Auts2* is a long and complex gene, and it has been suggested that various isoforms are produced by alternative (splicing and alternative) transcriptional start sites. They have been intensively analyzed in zebrafish (Kondrychyn et al., 2017) but have not yet been reported in great detail in mammals. The human *AUTS2* gene has two main transcripts, a full-length and a 3′ short transcript with an alternative transcription start site within exon 9 (Beunders et al., 2013), whereas mouse *Auts2* has a full-length and two 3′ short isoforms arising from exon 8 and 9 (Hori and Hoshino, 2017). In the developing mouse brain, AUTS2 is highly expressed in various brain regions including cerebral cortex, hippocampus, and cerebellum (Bedogni et al., 2010). The knockdown of zebrafish *auts2* by morpholino leads to the drastic reduction of brain size, especially in caudal regions including the midbrain and hindbrain as well as the cerebellum (Oksenberg et al., 2013), suggesting that AUTS2 is crucial for brain tissue development. We have previously reported that cytoplasmic AUTS2 regulates actin cytoskeletal rearrangements via Rho family small GTPases, Rac1 and Cdc42, to control neuronal migration and neurite formation in the cortical neurons of prenatal forebrains (Hori and Hoshino, 2017). In addition, another group showed that nuclear AUTS2 interacts with histone modifiers such as Polycomb group (PcG) protein complex PRC1 and histone acetyltransferase P300 and acts as a transcriptional activator (Gao et al., 2014). Moreover, we previously showed that AUTS2 restricts the number of excitatory synapses without affecting that of inhibitory synapses in the telencephalon (Hori et al., 2020). This function is elicited by nuclear AUTS2, because nuclear localizing, but not cytoplasmic-localizing, AUTS2 is able to rescue the corresponding synaptic abnormalities in the *Auts2*-knockdowned primary cultured hippocampal neurons.

In the cerebellar cortex, the expression of *Auts2* mRNA was reported to start in PCs from the early neurodevelopmental stages and is maintained through postnatal and adult stages (Bedogni et al., 2010). However, little is known with regard to the physiological roles of AUTS2 in PC development due to lack of studies on the consequences of *Auts2* gene deletion in the cerebellum. Moreover, the extent of AUTS2 contribution to the pathogenesis of psychiatric disorders associated with the cerebellum remains unclear. Because conventional homozygous *Auts2* knockout mice are neonatal lethal (Hori et al., 2014), it has been difficult to study the function of AUTS2 in the cerebellum at postnatal stages and adulthood.

In this study, we generated *Auts2* conditional knockout (cKO) mice by crossing *Auts2*^*flox*^ mice with *En1*^*Cre*^ mice to disrupt the *Auts2* locus in the cerebellum (Hori et al., 2014; Kimmel et al., 2000; Sgaier et al., 2007). In the cerebella of these cKO mice, exon 8 of the *Auts2* gene is deleted by the Cre recombinase activity, leading to the complete elimination of both full-length AUTS2 (~170 kDa) as well as the C-terminal AUTS2 short isoform variant 1 (S-AUTS2-Var1; ~88 kDa). In contrast, the C-terminal AUTS2 short isoform variant 2 (S-AUTS2-Var2; ~78 kDa) that originates from exon 9 is aberrantly increased, as has been observed in the cerebral cortex of *Auts2* global KO mice (Hori et al., 2014). *Auts2* cKO mice displayed drastic reduction of cerebellar size accompanied with reduced PC number. The maturation of PCs was delayed in *Auts2* cKO mice, in terms of dendrite morphology and gene expression profile. Although CF synapse development was impaired in the *Auts2* cKO mice, excessive PF synapse formation was observed. Furthermore, *Auts2* cKO mice exhibited abnormal motor function and vocal communication behavior. Thus, these findings suggest that *Auts2* is involved in the maturation and synaptogenesis of PCs during cerebellar development, contributing to vocal communication as well as motor function. Because vocal communication deficits were also observed in heterozygous *Auts2* cKO mice, this study should provide insight into understanding the pathology of human psychiatric disorders with *AUTS2* mutations, which are in general, heterozygous.

## Results

### AUTS2 Is Specifically Expressed in Purkinje Cells and Golgi Cells in the Postnatal Cerebellar Cortex

**Figure 1:**
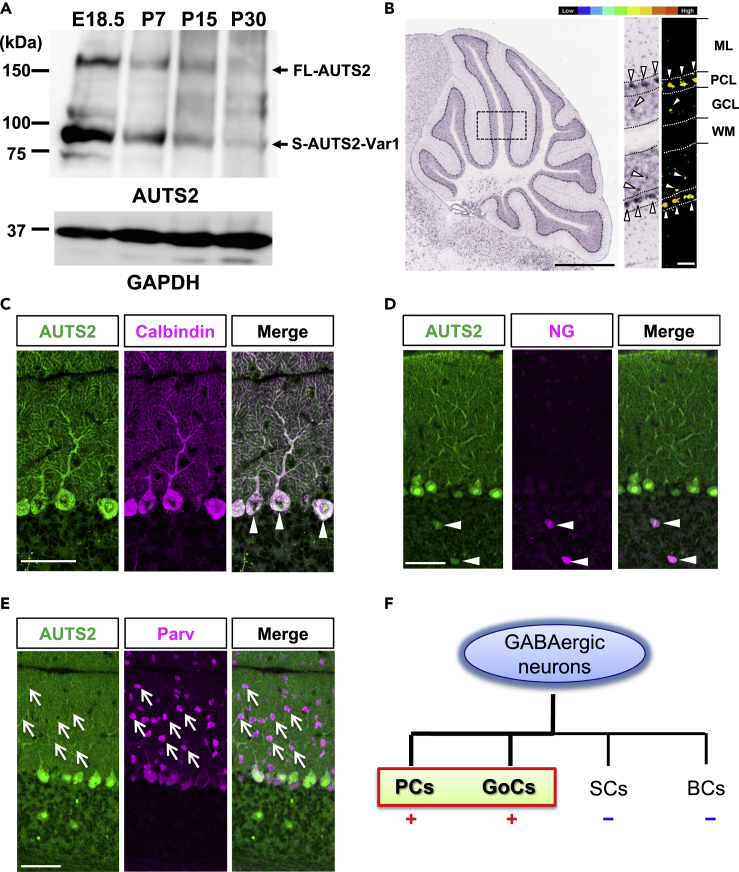
AUTS2 Expression in the Inhibitory Neurons in the Cerebellar Cortex (A) Expression of AUTS2 in the developing cerebellum. Arrows indicate the full-length (FL-AUTS2) or C-terminal short isoform variant 1 (S-AUTS2-Var1) of AUTS2 protein. (B) *In situ* hybridization for *Auts2* in P56 cerebellum (adapted from the Allen Brain Atlas, experiment #79904156). Arrowheads indicate the expression of *Auts2* mRNA. ML: Molecular layer, PCL: Purkinje cell layer, GCL: Granule cell layer, WM: White matter. Scale bar, 1 mm (left panel) and 100 μm (right panel). (C–E) Co-immunostaining of AUTS2 with inhibitory neuronal markers Calbindin (Purkinje cells), Neurogranin (NG; Golgi cells), and Parvalbumin (Parv; interneurons including stellate cells and basket cells at ML and Purkinje cells) in P25 cerebellar cortex. AUTS2 is expressed in Purkinje cells and Golgi cells (arrowheads in C and D), whereas there are no detectable signals in the molecular layer interneurons (arrows in E). Scale bars, 50 μm. (F) Summary diagram of AUTS2^+^ cells in inhibitory neurons in cerebellar cortex. PCs: Purkinje cells, GoCs: Golgi cells, SCs: stellate cells, BCs: basket cells. See also Figure S1.

To investigate the role of AUTS2 in postnatal cerebellar development, we examined the expression of AUTS2 in the cerebellum. Our previous study revealed that AUTS2 isoforms including the full-length (FL)-AUTS2 protein as well as the C-terminal short isoform variant 1 (S-AUTS2-Var1) are expressed in the cerebral cortex (Hori et al., 2014). Western blotting analysis with whole cerebellar lysates showed that FL-AUTS2 and S-AUTS2-Var1 are expressed at the late embryonic stage (E18.5), and expression gradually decreases throughout postnatal development, although still observed at postnatal day 30 (P30) (Figure 1A). Consistent with previous studies (Bedogni et al., 2010), *in situ* hybridization data from the Allen Brain Atlas (http://portal.brain-map.org) show that *Auts2* is highly expressed in PCs in adults (Figure 1B). In addition, we found that *Auts2* mRNA is also detected in certain cells in the granule cell layer (GCL) (Arrowheads in Figure 1B). Co-immunostaining of adult cerebellar tissues using the anti-AUTS2 antibody with cell-specific markers demonstrated that AUTS2 colocalized with calbindin, a marker for PCs (Figure 1C). Furthermore, immunofluorescence analyses on the developing cerebellar tissue sections postnatally revealed intense AUTS2 immunosignals in PCs equivalent to those observed in adult cerebellum (Figure S1), indicating that AUTS2 expression is maintained in PCs in development and adulthood. Within the PCs, AUTS2 is found in cell bodies including nuclei and dendrites (Figures 1C and S1). Consistent with *Auts2* mRNA expression, the immuno-signals for AUTS2 were also detected in the neurogranin-positive Golgi cells in the GCL (Figure 1D) (Singec et al., 2003). In contrast, AUTS2 was not detected in the parvalbumin-positive interneurons in the ML including stellate cells and basket cells (Figure 1E). These results suggest that AUTS2 is exclusively expressed in PCs and Golgi cells in the cerebellar cortex during postnatal development (Figure 1F).

### *Auts2* Conditional Knockout Mice Exhibit Defects in Cerebellar Development

**Figure 2:**
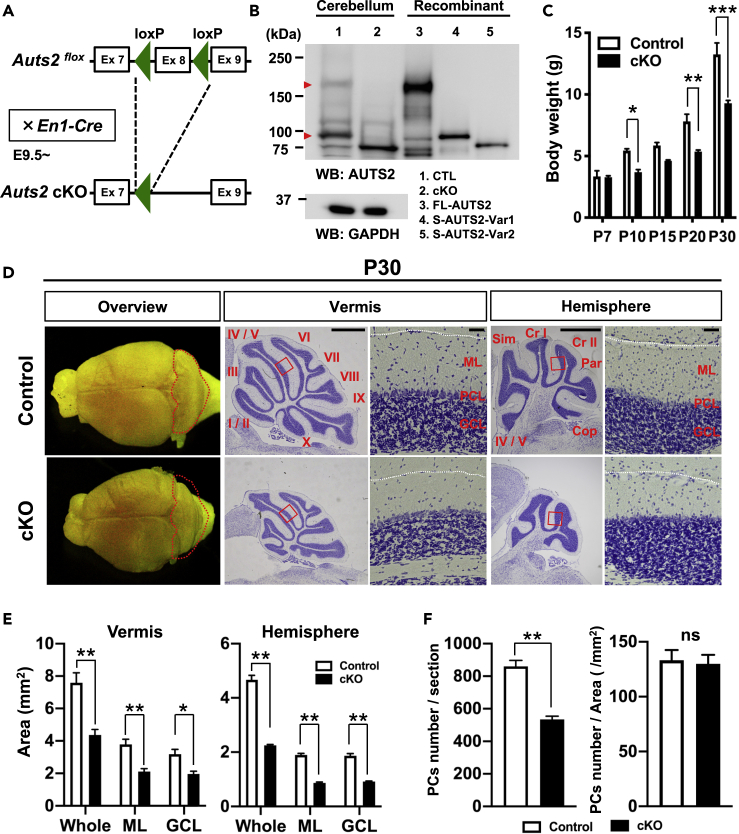
Cerebellar Hypoplasia in *Auts2* Conditional Knockout Mice (A) Schematics of the targeting strategy for *Auts2* conditional knockout (*Auts2* cKO) mice. Exon 8 of *Auts2* gene was conditionally deleted by crossing *Auts2-floxed* mice with *Engrailed-1*^*Cre/+*^ (*En1*^*Cre/+*^) mice. (B) Immunoblot for AUTS2 proteins in cerebellar lysates from *Auts2*^*flox/flox*^ (Control; CTL) and *En1*^*Cre/+*^;*Auts2*^*flox/flox*^ homozygotic cKO mice at P0. Immunoblot of lysates from HEK293T cells expressing the recombinant full-length AUTS2 (FL-AUTS2) and the C-terminal AUTS2 short variants (S-AUTS2-Var1 and Var2) are also shown. Full-length AUTS2 as well as the S-AUTS2-Var1 were completely eliminated in *Auts2* cKO homozygotic mutant cerebellum (red arrowheads), whereas the S-AUTS2-Var2 was alternatively increased. (C) Plot of body weights in control and *Auts2* cKO mice from P7 to P30. n = 2–7 mice. (D) Whole-mount images and Nissl-stained parasagittal sections in control and *Auts2* cKO mice at P30. The folia of vermis and hemisphere are indicated as roman numerals (I-X) and abbreviations (Sim: Simple lobule, Cr I and II: Crus I and II, Par: Paramedian lobule, Cop: Copula pyramidis). Higher magnification images of the boxed regions showing ML-PCL-GCL laminar structure. ML: Molecular layer, PCL: Purkinje cell layer, GCL: Granule cell layer. Scale bar, 1 mm and 50 μm. (E) Quantification of cerebellar areas including whole, molecular layer (ML), granule cell layer (GCL) in parasagittal sections of control and *Auts2* cKO mice at P30. n = 6 slices from 3 mice. (F) The number of PCs was decreased in the cerebellar vermis of *Auts2* cKO at P30 compared with the control, but the density of PCs was normal. n = 5 slices from 5 mice. Data are shown as mean ± SEM. ∗p < 0.05, ∗∗p < 0.01, ∗∗∗p < 0.001 by two-way ANOVA followed by Bonferroni's multiple comparisons test in (C), Mann-Whitney test in (E and F). See also Figures S2–S6.

We previously reported that homozygotes for the loss of function allele (*Auts2*^*del8*^) were neonatally lethal (Hori et al., 2014). To better understand the roles for AUTS2 in postnatal cerebellar development, we generated *Auts2* conditional KO (cKO) mice by crossing *Auts2*^*flox*^ with *En1*^*Cre*^ mice, in which exon 8 of *Auts2* can be specifically ablated in the rhombomere-1-derived brain area including the cerebellum from the mid-embryonic stages (E9.5~) (Figure 2A) (Kimmel et al., 2000; Sgaier et al., 2007). In this study, we analyzed the *En1*^*Cre*/+^;*Auts2*^*flox/flox*^ (homozygous *Auts2* cKO) and *Auts2*^*flox/flox*^ (control) mice unless otherwise noted. As previously observed in the cerebral cortices of *Auts2*^*del8*^ mutants (Hori et al., 2014), immunoblotting of cerebellar tissue extracts confirmed that in the *Auts2* cKO cerebella, both FL-AUTS2 and S-AUTS2-Var1 are eliminated, whereas S-AUTS2-Var2 that originates from exon 9 is abnormally increased (Figure 2B). To confirm the specificity of the AUTS2 antibody used in immunoblotting, we performed immunostaining using this antibody on prenatal cerebellum prepared from *Auts2*^*neo/neo*^ homozygous mutant mice, in which all AUTS2 isoforms were almost completely eliminated (Figure S2) (Hori et al., 2014). Immunofluorescence shows the expression of AUTS2 in RORα-positive Purkinje cells in WT cerebellum, whereas immuno-signals were almost completely absent in *Auts2*^*neo/neo*^ homozygotes (Figure S2), indicating the high specificity of this antibody for AUTS2. Moreover, quantitative PCR showed that excision of exon 8 within the *Auts2* mRNA was almost complete in the cerebella of *En1-Cre; Auts2*^*flox/flox*^, whereas exon 8 remained intact in the cerebral cortices of the same animals (Figure S3).

*Auts2* cKO mutants were viable but had a significant reduction in body weight or exhibited developmental delays (Figure 2C). At P30, cerebella isolated from the *Auts2* cKO mice were smaller than those of controls (Figure 2D). Sagittal cerebellar sections of *Auts2* cKO mice revealed a dramatic reduction in size of both hemispheres and vermis regions compared with control (Figure 2D). In addition, *Auts2* cKO mutants exhibited aberrant cerebellar cortical morphologies. Several lobules including lobe X, Crus I, and copula pyramidis were severely reduced in size or absent (Figure 2D). Sections of *Auts2* cKO cerebellar cortices revealed that although the basic laminar structure consisting of ML-PCL-GCL was normal (Figure 2D), the total areas including both ML and GCL were decreased by ~56% (Figure 2E). Furthermore, the number of PCs in *Auts2* cKO mice were significantly decreased, whereas the density of PCs was similar (Figures 2F and S4).

In addition to the cerebellum, *En1* is expressed in the caudal midbrain (Sgaier et al., 2007). Although there are no gross histological differences in the midbrain regions between *Auts2* cKO mice and controls (data not shown), we found that the number of dopaminergic midbrain neurons in the substantia nigra were slightly but significantly reduced in *Auts2* cKO mice compared with the control mice (Figure S5).

It is well established that the signaling factor Sonic Hedgehog (SHH), secreted from PCs, plays a key role for GC expansion during the cerebellar development (Dahmane and Ruiz i Altaba, 1999). To determine whether loss of *Auts2* leads to reduced SHH expression or impairment of SHH signaling in the developing cerebellum, we performed immunostaining for SHH and its downstream effector GLI1 on tissue sections from the developing cerebellum. Immunofluorescence on cerebellum from *Auts2* cKO mice at P7 showed that the SHH immunosignals in PCs were comparable with control (Figure S6A). We also detected the expression of GLI1 in the external granular layer (EGL) in both genotypes, suggesting that SHH signaling functions normally to activate the expression of the SHH downstream effector(s) in granule neuron precursors in the *Auts2* cKO cerebellum (Figure S6A). Furthermore, RT-qPCR analysis revealed no significant differences in the expression levels of these genes per cerebellar unit among the genotypes (Figure S6B). These results imply that the reduction of GCs in the *Auts2* mutants might be attributed to the reduction of SHH due to the diminished number of PCs. Taken together, these results suggest that AUTS2 is critical for cerebellar development.

### AUTS2 Regulates Dendritic Outgrowth of Purkinje Cells

**Figure 3:**
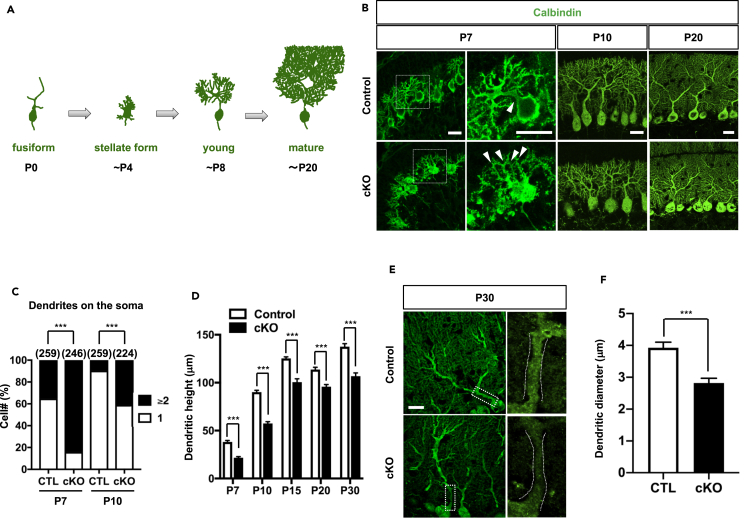
Loss of *Auts2* Induces Impaired Maturation of PCs (A) Schematics of PC morphology during the postnatal development. (B) Representative immunofluorescent images of Calbindin-positive PCs in lobule IV/V from P7 to P20 in control (upper panels) and *Auts2* cKO mice (lower panels). Arrowheads indicate dendrites on the soma. Scale bars, 20 μm. (C) Proportion of the number of primary dendrites formed on single PC soma in lobule IV/V at P7 and P10 in control (CTL) and *Auts2* cKO mice. n = 259 cells from 3 mice at P7 and P10 for control mice, and n = 246 cells from 3 mice at P7, n = 224 cells from 3 mice at P10 for *Auts2* cKO mice. (D) Measurement of dendrite lengths of PCs in lobule IV/V toward the pia surface during postnatal development. n = 12–15 cells from 3 mice for control and *Auts2* cKO mice. (E) Representative images of primary dendritic shafts of PCs in lobule IV/V labeled with Calbindin at P30 in control and *Auts2* cKO mice. Scale bar, 20 μm. (F) Reduced PC primary dendrite thickness of lobule IV/V in *Auts2* cKO mice. n = 12–14 cells from 3 mice. Data are shown as mean ± SEM. ∗p < 0.05, ∗∗p < 0.01, ∗∗∗p < 0.001 by Chi-squared test in (C), two-way ANOVA followed by Bonferroni's multiple comparisons test in (D), Mann-Whitney test in (F). See also Figure S7.

Among the AUTS2-positive cerebellar inhibitory neurons, PCs play a key role in the output of processed information and control of motor function. We therefore decided to focus on the roles of AUTS2 in PC development. In the P0 cerebellar cortex, postmigratory PCs initially display “fusiform” morphology with a few primitive apical dendrites (Figure 3A). They then transform into “stellate cells” by retracting primitive dendrites, which in turn, form multiple disoriented perisomatic dendrites by P4. During the next four days, these irregular dendrites are progressively regressed concomitantly with the occurrence of single stem apical dendrite (primary dendrite), and PCs enter the “young PC” stage by P8. Subsequently, PCs continue to extend dendrites and form highly refined branches, reaching maximal lengths by around P20 (Sotelo and Dusart, 2009). To investigate the dendritogenesis of PCs in *Auts2* cKO mice, we used calbindin. In the control cerebellar cortex at P7, the majority of PCs displayed typical “young PC”-like morphology, with a single thick stem dendrite and elaborated branches (Figures 3B and 3C). In contrast, most of the PCs in *Auts2* cKO mice at the same age appeared stellate cell-like in shape with more than 2 perisomatic dendrites (Figures 3B and 3C). By P10, although the proportion of the cells with young PC morphologies was increased to ~60% in *Auts2* cKO mice, higher numbers of PCs still exhibited stellate-like shapes compared with the controls (Figures 3B and 3C). These observations suggest that the pruning process of PC dendrites is impaired in *Auts2* cKO mice. Consistent with a reduction in the ML in *Auts2* cKO cerebellum (Figure 2E), we observed a reduction in dendritic outgrowth of PCs in *Auts2* cKO cerebellum throughout postnatal stages (Figure 3D). The diameter of the first segment of primary dendrites was significantly smaller in *Auts2* cKO PCs than that of control (Figures 3E and 3F).

We further examined whether the impairments in dendrite development of PCs in *Auts2* cerebellum is caused by the increased S-AUTS2-Var2 expression. Overexpression of S-AUTS2-Var2 in PCs in WT cerebellum did not, however, affect dendritic outgrowth nor its morphology (Figure S7), suggesting that the defects in dendritogenesis in *Auts2* cKO mutant PCs was not due to a gain-of function effect by increased S-AUTS2-Var2. Taken together, these results suggest that AUTS2 is involved in proper development of PC dendrites.

### Loss of *Auts2* Causes Abnormal CF and PF Synapse Formation in PCs

**Figure 4:**
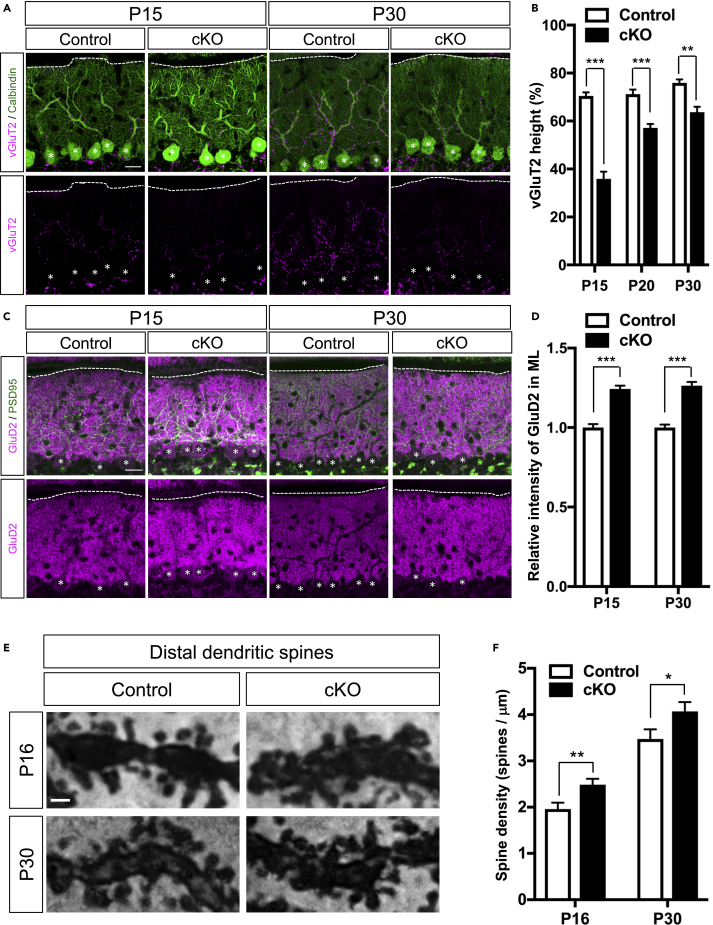
Delayed CF Translocation and Excessive PF Formation in *Auts2* Conditional Knockout Mice (A) Double immunostaining with calbindin (green) and climbing fiber (CF) synaptic marker VGlut2 (magenta) on the cerebellar lobule IV/V of control and *Auts2* cKO mice at P15 and P30. Scale bars, 20 μm. (B) Quantitative analysis of the ratio of VGlut2 height to the tip of PC dendrites of lobule IV/V in control and *Auts2* cKO cerebellum during P15-30. n = 12–15 cells from 3 mice. (C) Representative images of co-immunostaining with PSD-95 (green) and parallel fiber (PF) postsynaptic marker GluD2 (magenta) of lobule IV/V in the molecular layer (ML) of control and *Auts2* cKO mice at P15 and P30. Scale bars, 20 μm. (D) Increased immunofluorescence intensity levels of GluD2 in lobule IV/V of *Auts2* cKO mice at P15 and P30. n = 72–108 areas from 3 mice. (E) Representative images of the dendritic spines on distal PC dendrites in the Golgi-stained cerebellar lobule IV/V of control and *Auts2* cKO mice at P16 and P30. Scale bar, 1 μm. (F) The density of distal dendritic spines on PCs of lobule IV/V was increased in *Auts2* cKO mice at P16 and P30. n = 18–27 branches, 3 mice. Data are shown as mean ± SEM. ∗p < 0.05, ∗∗p < 0.01, ∗∗∗p < 0.001 by two-way ANOVA followed by Bonferroni's multiple comparisons test in (B), Mann-Whitney test or unpaired Student's t-test in (D and F). Dotted lines and asterisks indicate the pial surface of the ML and PC soma, respectively. See also Figures S8–S11.

We next investigated the function of AUTS2 in PC synapse formation. PCs receive excitatory synaptic inputs from CF neurons in the ION. The CF axon terminals from ION translocate upward from soma to primary dendrites of PCs, forming excitatory synapses (CF synapses). Immunohistochemistry of postnatal cerebellar sections with VGlut2, a marker for presynaptic terminals of the CFs, showed that, at P15, VGlut2-puncta traversed 70.45 ± 1.54% of the ML thickness in control cerebella, whereas they were found only in the deeper regions (35.93 ± 3.01%) of the ML in the *Auts2* cKO cerebella (Figures 4A and 4B). Although those VGlut2-puncta gradually translocated upward in the *Auts2* cKO cerebella as development proceeded, they never reached the level of the control mice at P30 (Figures 4A and 4B). Similarly, VGlut2-puncta in the ML at *Auts2* cKO cerebella were significantly decreased compared with the controls (Figures S8A and S8B). This suggests that development of CF synapses, particularly their translocation process, is delayed in *Auts2* cKO mice.

We next assessed the parallel fiber (PF) synapses by immunostaining with GluD2, a molecular marker for PF synapses in PC dendrites (Yamasaki et al., 2011). In contrast to the CF synapses, we observed that loss of *Auts2* resulted in an increase of the GluD2-immunoreactivities in the ML of *Auts2* cKO mice compared with those of control mice at P15 and P30 (Figures 4C and 4D). Likewise, high-magnification images in the ML showed that the density of GluD2-puncta was significantly higher in *Auts2* cKO mice (Figures S8C and S8D), suggesting that loss of *Auts2* leads to excessive PF synapse formation. Golgi staining also revealed that the dendritic spine density at the distal end of the PC dendrites was significantly increased in *Auts2* cKO mutants compared with controls at P16 and P30 (Figures 4E and 4F). Because the distal part of PC dendrites is predominantly occupied by PF synapses (Altman, 1972), the increased number of synapses in the *Auts2* cKO cerebella were regarded as PF synapses. These findings suggest that *Auts2* is required for normal development of CF synapses, while restricting the number of PF synapses. We confirmed that overexpression of S-AUTS2-Var2 in the PCs in WT cerebellum did not alter the translocation of CF synapses (Figure S9) as well as the number of dendritic spines at the distal end of PC dendrites (Figure S10), suggesting that the aberrant excitatory synapse development observed in *Auts2* cKO mutants is caused by loss of function of AUTS2.

It was previously reported that AUTS2 acts as a transcriptional regulator for neural development (Gao et al., 2014). Furthermore, our previous RNA-seq analysis showed that disruption of *Auts2* in mouse forebrains resulted in changes in global expression of genes associated with multiple aspects of neurodevelopment, including dendrite morphogenesis and synapse development (Hori et al., 2020). Quantitative PCR analysis showed no significant changes in the expression levels of *RORα* (Figure S11A) (Takeo et al., 2015). Meanwhile, we observed the downregulation of *Cacna1a*, which reportedly regulates the excitatory synapse formation in PCs, in the *Auts2* cKO mice (Figure S11A) (Hashimoto et al., 2011; Miyazaki et al., 2004, 2012). Furthermore, we verified that the intensity of CaV2.1 (a product of *Cacna1a* gene) immunostaining, is markedly reduced in *Auts2* cKO PCs (Figures S11B and S11C). These results imply that AUTS2 may be involved in CF and PF synapse development by regulating the expression of synaptic genes, such as *Cacna1a*.

### Purkinje-Cell-Specific *Auts2* Knockdown Impairs Excitatory Synapse Functions

**Figure 5:**
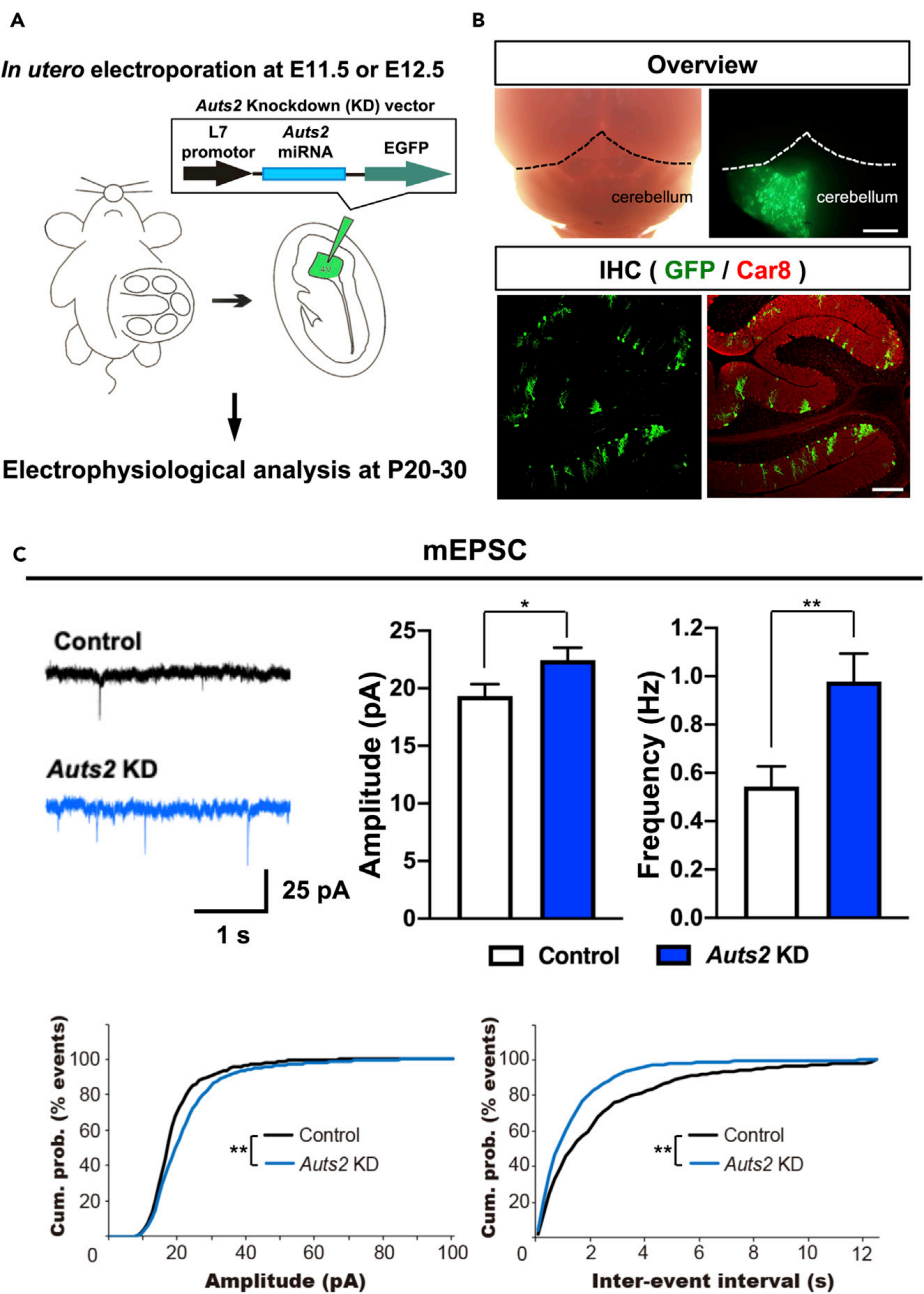
Knockdown of *Auts2* in PCs Exhibits Enhanced Excitatory Synaptic Transmission (A) Schematic diagrams indicate the knockdown (KD) experiments of *Auts2* with PC-specific expression vector. (B) Whole-mount and immunohistochemical images showing the successful introduction of *Auts2*-KD vector into PCs. EGFP-positive cells are co-labeled with a PC marker, Car8 (red). Scale bar; 1 mm (upper), 200 μm (lower). (C) *Auts2*-KD PCs enhance amplitude and frequency of mEPSC at P20-30. Panels show representative traces (upper left) and summary graphs of the mEPSC amplitude and frequency (upper right). Bottom, cumulative probability distributions of mEPSC amplitudes (left) and inter-event interval (right) in control and *Auts2*-KD PCs. n = 11 cells, 6 mice for control and n = 13 cells, 5 mice for *Auts2* KD. Data are shown as mean ± SEM. ∗p < 0.05, ∗∗p < 0.01, by unpaired student t-test in bar plots, Kolmogorov-Smirnov test in cumulative frequency plots. See also Figures S12–S14.

Next, we performed an electrophysiological analysis to investigate the loss-of-function effects of *Auts2* on PCs of interest. To evaluate the cell-autonomous effects of *Auts2* inactivation on the synaptic transmission properties of PCs, we introduced a vector expressing EGFP and *Auts2*-targeted microRNA (miRNA) driven by the PC-specific L7 promoter into PCs by *in utero* electroporation at E11.5-12.5 (Figure 5A). This miRNA was confirmed by western blotting to successfully downregulate the expression of both FL-AUTS2 and C-terminal short isoforms (Figure S12). Immunohistochemical analysis revealed that EGFP-positive cells were co-labeled with the PC marker, Car8 (Figure 5B) (Patrizi et al., 2008). We subsequently performed whole-cell patch-clamp recordings in EGFP-positive or -negative PCs from acute cerebellar slices at P20-30. To examine the basic properties of overall synaptic function in PCs targeted with the *Auts2*-knockdown (KD) vector, we measured the miniature excitatory and inhibitory postsynaptic currents (mEPSCs and mIPSCs, respectively). The amplitude and frequency of mEPSCs were significantly increased in EGFP-positive *Auts2*-KD PCs compared with non-transfected PCs (EGFP-negative), whereas those of mIPSCs were not affected (Figures 5C and S13). The effect of *Auts2*-KD on mEPSCs was sufficiently restored by co-transfection of an RNAi-resistant FL-AUTS2 (*Auts2*-Res) construct, which successfully excluded off-target effects of the targeting miRNA (Figure S14). This suggests that FL-AUTS2 has the ability to regulate excitatory synaptic transmission in PCs.

**Figure 6:**
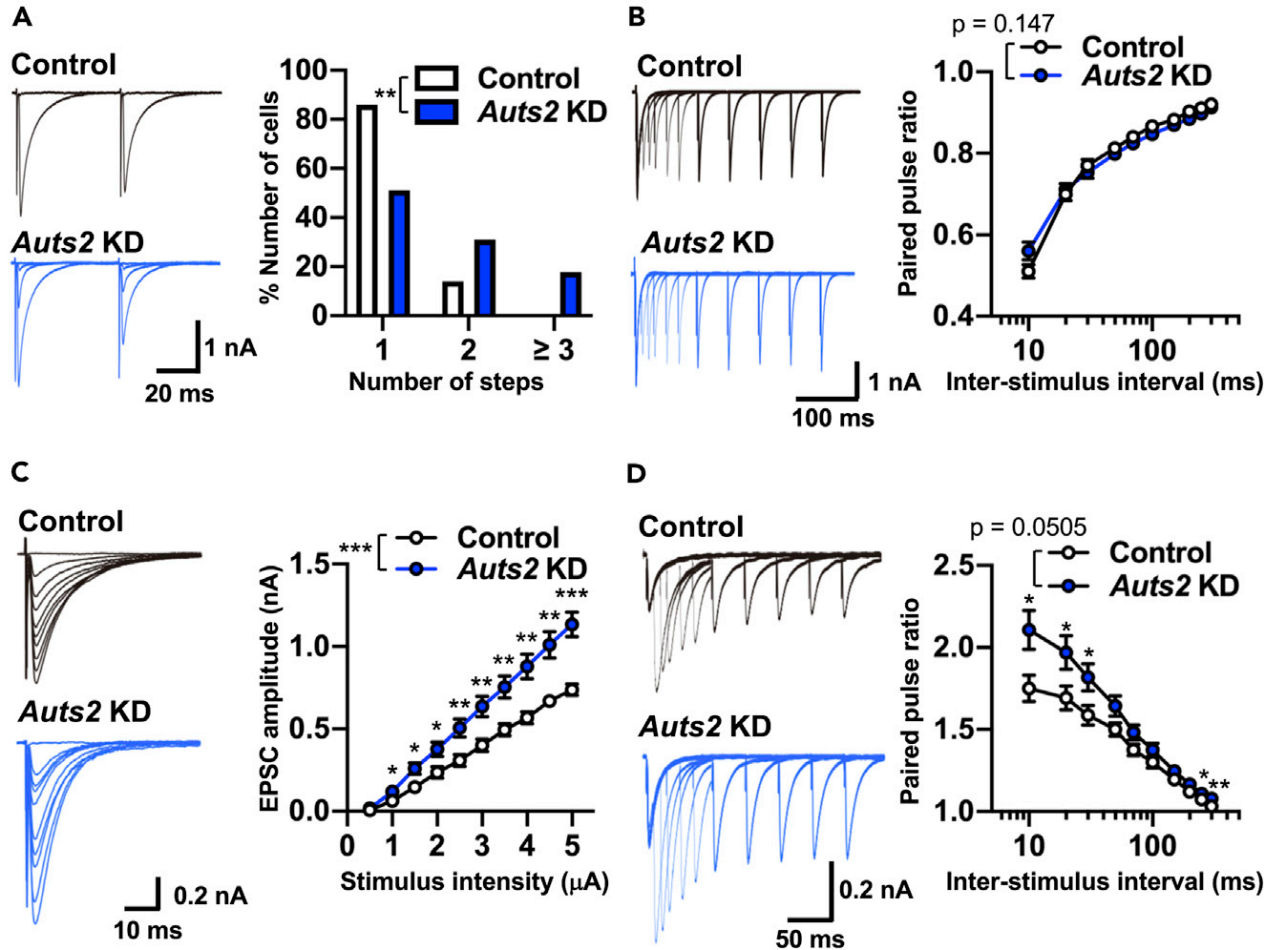
Knockdown of *Auts2* in PCs Impairs CF Synapse Elimination and PF Synaptic Transmission (A) Sample traces of CF-EPSCs (left) and frequency distributions of the number of CFs innervating each PC (right) for *Auts2*-KD (blue) and control (white) PCs during P21-P30. n = 37 cells, 3 mice for control and n = 33 cells, 3 mice for *Auts2* KD. (B) Normal paired-pulse ratio of CF-EPSCs measured at increasing inter-stimulus intervals in control and *Auts2*-KD PCs at P20-30 (left, representative traces; right, summary plots). n = 13 cells, 3 mice for control and n = 26 cells, 3 mice for *Auts2* KD. (C) Impaired input-output relationship of PF-EPSCs in *Auts2*-KD PCs at P20-30. (left, representative traces; right, summary plots). n = 14 cells, 6 mice for control and n = 17 cells, 6 mice for *Auts2* KD. (D) Impaired paired-pulse ratio of PF-EPSCs in *Auts2*-KD PCs at P20-30 (left, representative traces; right, summary graph). n = 13 cells, 6 mice for control and n = 16 cells, 6 mice for *Auts2* KD. Data are shown as mean ± SEM. ∗p < 0.05, ∗∗p < 0.01, ∗∗∗p < 0.001, by Mann-Whitney U test in A, two-way ANOVA with Tukey's post hoc analysis in B-D. See also Figure S15 and Table S1.

Next, we recorded climbing fiber-evoked EPSCs (CF-EPSCs) to test whether loss of *Auts2* function in PCs affected CF synapse function. During early postnatal stages, each PC cell body receives multiple CF presynaptic inputs innervating from ION neurons. Subsequently, a single CF is selectively strengthened to form CF synapses, translocating along the primary dendrites of the PC while the other surplus CFs are eliminated (Kano et al., 2018). We moved the stimulation electrode systematically around the PC soma under recording and increased the stimulus intensity gradually at each stimulation site. The recording highlighted that more than 80% of PCs in WT mature cerebellum show a single-step response to an evoked CF input, indicating that the majority of PCs receive a single CF input (Figure 6A). By contrast, around half of *Auts2*-KD PCs received multiple CF inputs, compared with only 14% of non-transfected PCs (Figure 6A), indicating that loss of *Auts2* impairs the elimination of surplus CFs in PCs. We also examined the functional differentiation of multiple CF inputs by calculating two parameters, the disparity ratio and disparity index (Hashimoto and Kano, 2003). The disparity ratio and index of *Auts2*-KD PCs were similar to non-transfected PCs (Figure S15). Furthermore, we tested whether *Auts2*-KD PCs exhibited abnormal electrophysiological properties of CF-EPSCs. We observed a longer 10%–90% rise time and shorter decay time, but a normal amplitude in the total CF-EPSCs in *Auts2*-KD PCs (Table S1). Indeed, there was no difference in the extent of paired-pulse depression at inter-pulse intervals from 10 to 300 msec between *Auts2*-KD and non-transfected PCs, indicating that the release probability of CF synapses was normal in *Auts2*-KD PCs (Figure 6B). We concluded that AUTS2 is required for the selection of a single CF to innervate PC by eliminating other CFs.

Subsequently, we examined the electrophysiological properties of parallel fiber-evoked EPSCs (PF-EPSCs). The input-output curve shows that PF-EPSCs were markedly increased in *Auts2*-KD PCs (Figure 6C), consistent with the increased number of PF synapses in *Auts2* cKO mice observed by immunohistochemistry and Golgi staining (Figures 4C–4F, S8C, and S8D). Interestingly, the extent of paired-pulse facilitation was greater in *Auts2*-KD PCs, suggesting that AUTS2 is also involved in the release probability of PF synapses (Figure 6D). Taken together, these results suggest that in PCs, AUTS2 is required for the regulation of PF synaptic function.

### *Auts2* cKO Mice Display Motor Dysfunction and Impaired Vocal Communication

**Figure 7:**
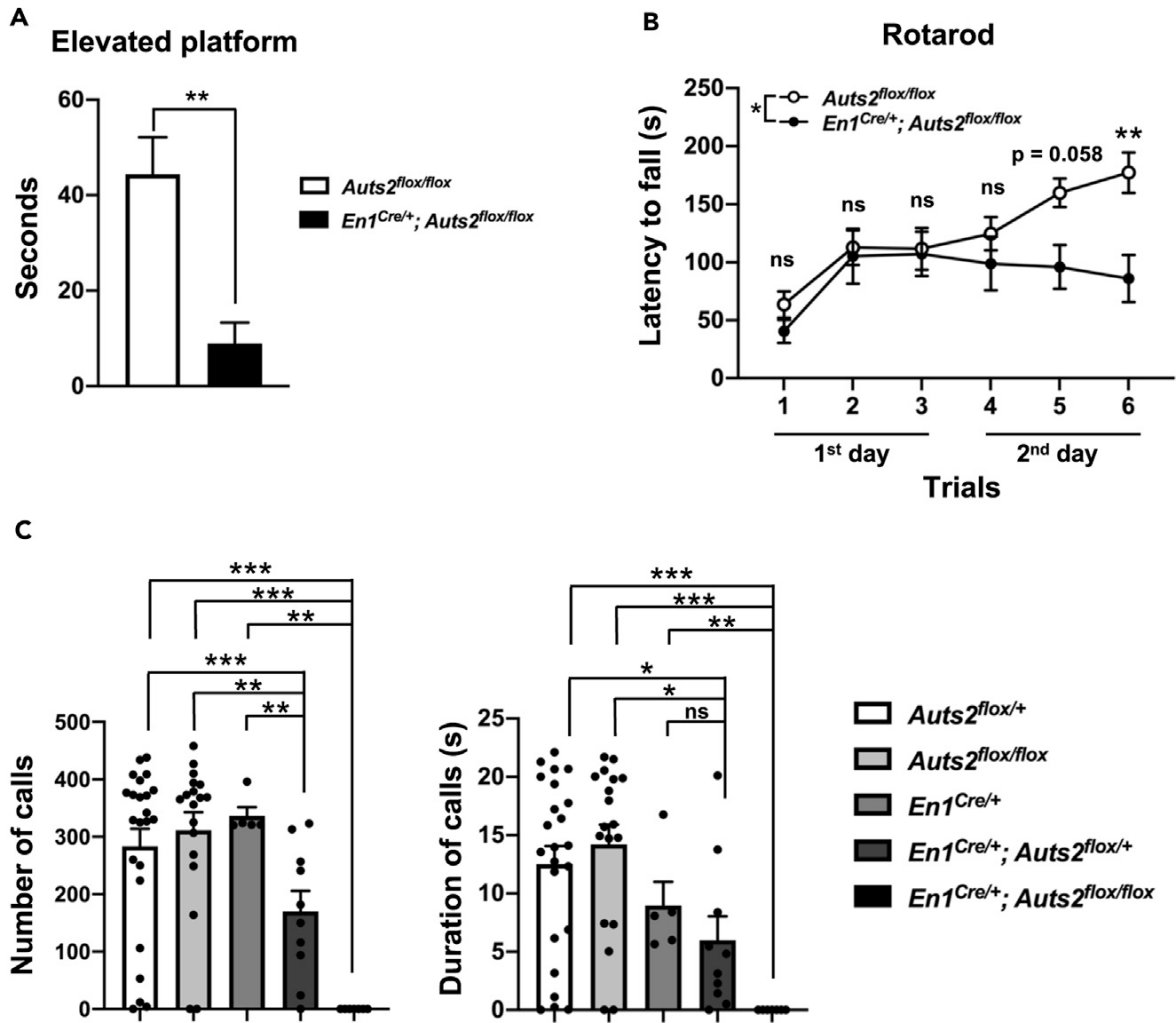
Motor Dysfunction and Impaired Vocal Communication in *Auts2* cKO Mice (A) *Auts2* cKO mice exhibit motor abnormality in elevated platform test. n = 8 mice. (B) *Auts2* cKO mice show impaired motor learning in an accelerating rotarod test. n = 13 mice for control mice and 9 mice for *Auts2* cKO mice. (C) USV recordings show the severe impairments of vocal communication in *Auts2* cKO mice. n = 23 mice for *Auts2*^*flox/+*^ mice, 18 mice for *Auts2*^*flox/flox*^ mice, 5 mice for *En1*^*Cre/+*^ mice, 10 mice for *En1*^*Cre/+*^*; Auts2*^*flox/+*^ mice, 7 mice for *En1*^*Cre/+*^*; Auts2*^*flox/flox*^ mice. Data are shown as mean ± SEM. ∗p < 0.05, ∗∗p < 0.01, ∗∗∗p < 0.001 by Mann-Whitney test in (A and C), two-way ANOVA followed by Bonferroni's multiple comparisons test in (B).

Next, we performed several behavioral analyses on *Auts2* cKO mice. In the elevated platform test (Alvarez-Saavedra et al., 2014), mice were placed on a small round elevated platform and the time for which mice remained on the platform was recorded (Figure 7A). *Auts2* cKO mice exhibited a significant decrease in the length of time able to keep their balance on the platform compared with control mice, suggesting that *Auts2* cKO mice had defects in motor control (Figure 7A). We further examined the motor coordination and motor learning with the accelerating rotarod test. Control and *Auts2* cKO mice behaved similarly in the three trials during the first day of testing (Figure 7B). However, on the second day, although the motor performance of the control mice improved, *Auts2* cKO mice did not show such improvement, suggesting that *Auts2* cKO mice had abnormalities in motor learning rather than in motor coordination (Figure 7B).

Subsequently, we measured ultrasonic vocalizations (USVs) of adult male mice. Male mice use courtship USVs when exposed to female mice. However, both the number and duration of calls were eliminated in homozygous *Auts2* cKO males (*En1*^*Cre/+*^*;Auts2*^*flox/flox*^) (Figure 7C). This suggests that AUTS2 expression in the cerebellum (or at least in the rhombomere 1 region) is critically required for male courtship USVs. Interestingly, the number and duration of calls were significantly reduced even in heterozygous *Auts2* cKO males (*En1*^*Cre/+*^*;Auts2*^*flox/+*^), suggesting that loss of one *Auts2* allele leads to communication deficits. This is very intriguing, because most patients with *AUTS2* mutations are heterozygotes for this gene.

## Discussion

In this study, we showed that specific ablation of *Auts2* in the cerebellum resulted in various structural, physiological, and behavioral abnormalities. AUTS2 has been reported to have two distinct molecular and cellular functions in neural development. We have previously reported that AUTS2 acts in cytoplasm to regulate actin cytoskeleton by controlling Rho family GTPases, such as Rac1 and Cdc42 (Hori et al., 2014). Other groups demonstrated that nuclear AUTS2 functions to regulate the transcriptional activity of genes as a component of PRC1 (Gao et al., 2014; Russo et al., 2018). Although we cannot fully conclude whether the abnormalities observed in *Auts2* cerebellar cKO mice were caused by loss of function of either cytoplasmic or nuclear AUTS2, we believe that most phenotypes, especially anatomical abnormalities including the reduced cerebellar size, delay of PC maturation as well as aberrant synapse development, might be resulted from loss of nuclear function of AUTS2, by the reasons described below. However, because cytoplasmic AUTS2 can also regulate the cellular morphology via cytoskeletal rearrangements (Hori et al., 2014), it may be possible that abnormal dendrite shapes, at least in part, are caused by loss of cytoplasmic AUTS2 function.

### The Reduced Cerebellar Size in *Auts2* cKO Mice

The *Auts2* cKO mice exhibit a significant reduction in cerebellum size. The volume of the cerebellum is largely determined by the number of GCs that make up the bulk of the cells. Since granule cell precursors (GCPs) proliferate and survive with the support of SHH secreted from PCs (Dahmane and Ruiz i Altaba, 1999), the number of GCs may also be defined by the amount of SHH released from PCs. In the *Auts2* cKO mice, the absolute number of PCs was greatly decreased (Figures 2F and S4), whereas the amount of SHH expressed in the individual PCs did not seem to be altered between *Auts2* cKO mice and the controls (Figure S6). These results imply that the reduction of GCs in *Auts2* cKO mice may be attributed to the diminished number of PCs, resulting in a decrease in the amount of SHH in the cerebellum. During embryonic and postnatal development, we did not find increased apoptosis of PCs (data not shown). Therefore, we believe that PC production from the cerebellar ventricular zone may be reduced in *Auts2* cKO mice, although we do not have any direct evidence. Previous *in situ* hybridization analysis showed that the cerebellar ventricular zone expresses *Auts2* (Bedogni et al., 2010), and, moreover, recent single-cell RNA-sequencing analyses revealed that *Auts2* is expressed in a subpopulation of neural progenitors in both cerebral cortex and cerebellar primordium (Carter et al., 2018; Telley et al., 2019). *In vitro* analyses using mouse embryonic stem cells also demonstrated that the AUTS2-PRC1 complex is critical for neuronal differentiation (Russo et al., 2018). These findings imply that AUTS2 may be involved in production of PCs from the ventricular zone, although that issue is not the focus of this study. As to the decreased size of *Auts2* cKO cerebellum, we cannot rule out the possibility that AUTS2 intrinsically regulates the proliferation of granule cells. Although we did not detect significant levels of AUTS2 protein in the differentiated granule cells with our immunohistochemical conditions, other groups have reported that *Auts2* mRNA is weakly expressed in the neural progenitor cells at the rhombic lip and external granular layer (EGL) of cerebellar primordium (Bedogni et al., 2010). *Auts2* cKO mice crossed with Cre lines with more restricted expression, such as *Atoh1*-Cre (Fujiyama et al., 2009), will be useful for future studies to explore the role of AUTS2 in cerebellar development.

### The Involvement of AUTS2 in PC Maturation

The dendrite morphologies of *Auts2* cKO PCs seemed immature for their developmental ages. The mutant PCs tended to possess multiple primary dendrites on a single soma at P7 and P10 when control PCs usually harbored a single trunk dendrite at those stages. Dendrite height within the ML was also lower for the mutant PCs. These findings suggest *Auts2* cKO PCs are immature for their developmental stages. Because AUTS2 can regulate gene expression as a component of PRC1 (Gao et al., 2014), it is possible that AUTS2 directly or indirectly upregulates genes relevant for PC maturation. Those genes related to PC maturation may regulate PC dendrite development, and their reduced expression may account for the immature dendrite morphology of the *Auts2* cKO PCs. Alternatively, it is also possible that the dendrite morphology of PCs is regulated by cytoplasmic AUTS2. In general, the dendritic morphogenesis is strictly controlled by a variety of cytoskeletal proteins and their regulators. Among them, Rho-family small GTPases such as Cdc42 and Rac1 play pivotal roles in cytoskeletal reorganization during dendrite formation in neurons (Donald et al., 2008; Luo et al., 1996; Puram and Bonni, 2013). We previously reported that the cytoplasmic AUTS2 activates Rac1 via the Rac-GEF, P-Rex1, and Elmo2/Dock180 complexes while downregulating Cdc42 activities via Intersectin 1 and 2. AUTS2-Rac1 signaling is crucial for proper neurite outgrowth and branch formation in cerebral cortical neurons (Hori et al., 2014), implying that AUTS2 may regulate the dendritic morphogenesis of PCs using a common molecular machinery to regulate actin cytoskeleton.

### The Involvement of AUTS2 in Synapse Development on PCs

Previous studies indicated that AUTS2 is involved in various neurobiological functions ranging from neuronal proliferation, differentiation as well as neuronal migration and neuritogenesis. Our histological and electrophysiological analyses in this study revealed that AUTS2 is also required for proper synapse formation in PCs. During early postnatal stages, multiple CFs initially innervate a single PC soma, and one single CF is selectively strengthened and begins to form excitatory CF synapses on the PC dendrites, whereas the remaining redundant CF synapses are subsequently eliminated (Kano et al., 2018). PCs also receive an excitatory afferent from PFs of granule cells. PFs compete with CFs to form defined synapse territories on PC dendrites, and PF synaptic activity plays an important role in the pruning of surplus CFs. These CF refinement processes are highly regulated by various synaptic molecules. Among them, *Cacna1a*, a gene encoding P/Q-type voltage-dependent Ca^2+^ channel (also called CaV2.1), plays pivotal role in CF elimination and PF synapse boundary formation during postnatal development (Hashimoto et al., 2011; Miyazaki et al., 2004, 2012). Similar to the synaptic phenotypes in *Auts2* cKO mice, PCs lacking *Cacna1a* exhibit increased PF innervation as well as impaired CF translocation. qPCR and immunohistochemistry revealed that the expression of several synaptic molecules including CaV2.1/*Cacna1a* was decreased in *Auts2* cKO cerebellum. These results raise the possibility that nuclear AUTS2, as a component of PRC1, may participate in CF and PF synapse elimination/formation by regulating the expression of synaptic genes, such as *Cacna1a*. There are few studies reporting the involvement of *Cacna1a* in the dendrite morphogenesis of PCs. Other yet unidentified genes downstream of AUTS2 may also play important roles.

In *Auts2* cKO mice, excessive numbers of dendritic spines were formed in the distal region of PC dendrites. Consistent with this, downregulation of *Auts2* in PCs leads to the enhancement of PF-dependent excitatory neurotransmission. We previously observed that *Auts2* mutant mice exhibited increased spine formation in the forebrain, leading to the enhancement of excitatory synaptic inputs (Hori et al., 2020). A similar phenotype was observed in *Auts2* cKO cerebellum; dendritic spine numbers as well as excitatory inputs were increased without affecting inhibitory inputs in PCs. Because most of the dendritic spines and excitatory inputs we observed should correspond to PF synapses, AUTS2 may also function to restrict the number of PF synapses via its action in the cell nuclei, as was reported for the telencephalon (Hori et al., 2020).

### The Involvement of Cerebellar *Auts2* in Motor Function and Social Communication

The cerebellar neural circuit is well-known to be critical for motor coordination as well as motor learning (Apps and Garwicz, 2005). The vestibulocerebellar tract, which projects to lobules IX and X of the nodular cerebellum, carries information for balance (Maklad and Fritzsch, 2003; White and Sillitoe, 2013). We observed that loss of *Auts2* resulted in a reduction in cerebellar size, particularly of cerebellar subregions such as lobe X, Crus I, and copula pyramidis. Consequently, *Auts2* cKO mice displayed impaired motor control of balance as well as motor learning. These findings raise the possibility that dysgenesis of lobule X observed in *Auts2* cKO cerebellum contributes to the impairment in motor control.

Emerging evidence indicates that activation of PCs by the CF inputs drives motor skill learning such as vestibulo-ocular reflex (VOR) (Nguyen-Vu et al., 2013), whereas disruption of genes involved in synaptic transmission as well as intrinsic calcium signaling in PCs leads to impairment of motor learning (Aiba et al., 1994; Chen et al., 1995; Miyata et al., 2001). AUTS2 potentially regulates the expression of some synapse-related genes such as *Cacna1a*, which may participate in synapse formation required for motor function and learning.

It has previously been reported that loss of GCs or their synaptic functions resulted in behavioral abnormalities, such as motor incoordination and learning defects (Iskusnykh et al., 2018; Sathyanesan et al., 2018; Yoo et al., 2014). It is therefore difficult to distinguish whether the abnormal behaviors observed in this study were caused by the decreased cerebellar size or abnormal synapse formation, although we believe that both morphological alterations might affect behavioral abnormalities.

Recent studies highlighted the important roles for the cerebellum in higher cognitive functions, such as rewarding, social interaction, and social communication in addition to typical motor functions (Carta et al., 2019; Tsai et al., 2012). For example, the transcription factor *FOXP2* (forkhead box P2), is involved in speech in humans, and disruption of *Foxp2* in mice results in cerebellar abnormalities and an absence of vocalization, suggesting an association of the cerebellum with vocal communication (Fujita et al., 2008; Lai et al., 2001; Shu et al., 2005; Usui et al., 2017). Interestingly, Crus I was recently highlighted as a region of the cerebellum linked to cognition, social interaction, and language processing in both rodents and humans (Sokolov et al., 2017; Stoodley et al., 2017). Hence, dysgenesis of Crus I region might be responsible for impairment of vocal communication in *Auts2* cKO mice. Previous clinical studies reported that some individuals with *AUTS2* mutations display microcephaly, motor delay, and speech delay (Amarillo et al., 2014; Sengun et al., 2016). Cerebellar ablation of *Auts2* gene in mice results in a smaller cerebellum and the impairment of vocal communication. Interestingly, impairment of vocal communication was also observed in heterozygous *Auts2* cKO mice. Because most patients carry heterozygous *AUTS2* mutations, we believe heterozygous *Auts2* cKO mice and patients with *AUTS2* mutations may share a common pathology as to communication deficits.

In addition to the cerebellum, the midbrain is also involved in motor control, motivation, and reward behaviors as well as addiction through the dopaminergic neuron system (Hegarty et al., 2013). Emerging studies show that the blockade of the dopamine signaling pathway with dopamine receptor antagonists impairs motor learning (Beeler et al., 2012). Moreover, optogenetic activation of the midbrain dopaminergic neurons induces positive affective USVs in rats (Scardochio et al., 2015), whereas the emission of rat USVs can be repressed by dopamine antagonists (Brudzynski et al., 2012). In mature brains, a large population of dopaminergic neurons reside in the substantia nigra (SN) or ventral tegmental area (VTA) of the ventral midbrain (Hegarty et al., 2013). These dopaminergic neurons arise from the *En1*-derived precursor cells in rhombomere 1 during embryonic stages (Zervas et al., 2004). Bedogni et al. reported that *Auts2* expression is found in TH-positive dopaminergic neurons in the SN and VTA in the midbrain (Bedogni et al., 2010), suggesting that *Auts2* is also disrupted in these neurons in *Auts2* cKO mice. Intriguingly, we found that the TH-positive dopaminergic neurons were reduced in *Auts2* cKO midbrains in adult mice (Figure S5), implying that the potential defects of the dopaminergic pathways in the midbrains may also contribute to the behavioral abnormalities in *Auts2* cKO mice. Interestingly, Oksenberg et al. demonstrated by ChIP-sequencing analysis that AUTS2 binds to the promotor region of a Parkinson disease susceptibility gene, *Uchl1,* in the mouse (Oksenberg et al., 2014). Although further investigations are required to assess how loss of *Auts2* reduces dopaminergic neurons in the midbrain, our findings provide insights into a potential role of AUTS2 in the onset or progression of Parkinson disease or extrapyramidal disorder.

We previously performed behavioral analyses on two types of *Auts2* KO mice. In *Auts2*^*neo/+*^ mutants, the expression of all isoforms was reduced approximately by 50%. In the other (*Auts2*^*del8/+*^), the expression of FL-AUTS2 and S-AUTS2-Var1 was halved, whereas that of S-AUTS2-Var2 was increased. Interestingly, we observed distinct behavioral abnormalities in social interaction, anxiety, and prepulse inhibition, whereas no difference was detected in USVs. These observations suggested that abnormal overexpression of S-AUTS2-Var2 caused the behavioral abnormalities in those assays, and, therefore, we cannot exclude the possibility that aberrant expression of S-AUTS2-Var2 may also affect the results of behavioral abnormalities observed in this study.

This is the first investigation, to our knowledge, of the role of AUTS2 in the cerebellar development and function. The pathological mechanisms underlying how defects of cerebellar development caused by loss of AUTS2 function contribute to the psychiatric illnesses remain unclear. Further examination using our *Auts2* cKO mice will help to understand the pathological insights into the neurological disorders caused by *AUTS2* mutations.

### Limitations of the Study

Using western blotting, we demonstrated that the expression of FL-AUTS2 and S-AUTS2-Var1 proteins was eliminated in *Auts2* cKO cerebella, whereas that of S-AUTS2-Var2 was increased. Because immunostaining indicated that AUTS2 is expressed only in PCs and Golgi cells, such isoform changes may take place in PCs and Golgi cells in the *Auts2* cKO cerebella. However, it is difficult to confirm such isoform alteration with immunohistochemistry, because currently available AUTS2 antibodies mostly target the C-terminal region that is shared among all isoforms. Future studies to address the expression and function of each AUTS2 isoform in the cerebellum will require the development of better antibodies. With respect to behavioral abnormalities and weight loss, defects in cells other than PCs, namely Golgi and midbrain cells, may be involved. However, an miRNA study that reduced AUTS2 specifically in Purkinje cells resulted in abnormal electrophysiological responses in Purkinje cells, suggesting that impairments of PC maturation and synaptogenesis may likewise be caused by loss of AUTS2 in PCs.

Previously, we demonstrated that the impairments of neurite outgrowth in *Auts2*^*del8/del8*^ cortical neurons were sufficiently restored by FL-AUTS2 (Hori et al., 2014). Furthermore, aberrant spine formation in the *Auts2*-knockdown (KD) hippocampal neurons were also rescued by FL-AUTS2 but not the AUTS2 short isoforms (Hori et al., 2014). Although we did not assess the effects of loss of S-AUTS2-Var1 for PC dendrite and synapse development, these earlier observations suggest that FL-AUTS2 may regulate dendritic morphogenesis of PCs as well as the development of excitatory synapses on PCs.

The electrophysiological abnormalities in miRNA-introduced PCs were presumably caused by the loss of AUTS2 protein in a cell autonomous manner. However, we cannot exclude the possibility that they may be caused by some non-cell autonomous effects, because miRNA can occasionally be introduced into adjacent PCs.

In the behavioral tests, *Auts2* cKO mice exhibited abnormalities in the elevated platform test and rotarod test, which suggests impairments in motor coordination and learning. However, there remains the possibility that such abnormalities can also be caused by impaired emotional conditions, such as attention deficit or decreased anxiety.

### Resource Availability

#### Lead Contact

Further information and requests for resources should be directed to and will be fulfilled by the Lead Contact, Mikio Hoshino (hoshino@ncnp.go.jp).

#### Materials Availability

All unique materials generated from this study are available from the Lead Contact with a complete Materials Transfer Agreement.

#### Data and Code Availability

The datasets or codes in this study are available from the Lead Contact on request.

## Methods

All methods can be found in the accompanying Transparent Methods supplemental file.

## Supplemental Information

Document S1. Transparent Methods, Figures S1–S15, and Table S1
