# Supplementary material for: AUTS2 Governs Cerebellar Development, Purkinje Cell Maturation, Motor Function and Social Communication
Source: iScience. 2020 Nov 18;23(12):101820. doi: 10.1016/j.isci.2020.101820 (PMC7708818; doi:10.1016/j.isci.2020.101820)
Supplement: Document S1. Transparent Methods, Figures S1–S15, and Table S1 [file mmc1.pdf]

iScience, Volume *23*

Supplemental Information

AUTS2 Governs Cerebellar Development, Purkinje Cell Maturation, Motor Function and Social Communication

Kunihiko Yamashiro, Kei Hori, Esther S.K. Lai, Ryo Aoki, Kazumi Shimaoka, Nariko Arimura, Saki F. Egusa, Asami Sakamoto, Manabu Abe, Kenji Sakimura, Takaki Watanabe, Naofumi Uesaka, Masanobu Kano, and Mikio Hoshino

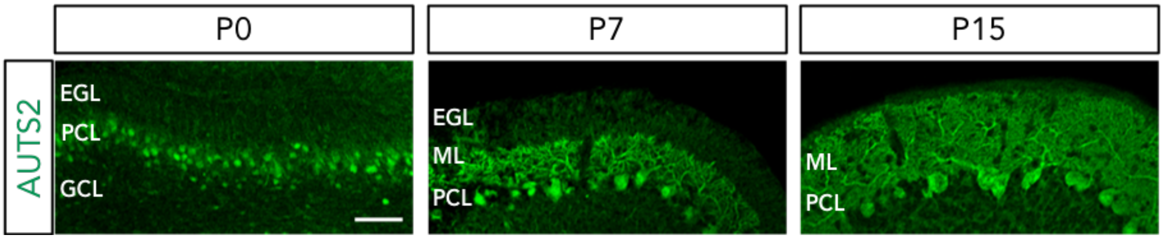

Figure S1. AUTS2 expression in PCs during postnatal cerebellar development, Related to Figure 1.

Representative images of the PCs stained with anti-AUTS2 antibody in WT developing cerebellum at P0, 7 and 15. Scale bar, 50 μm.

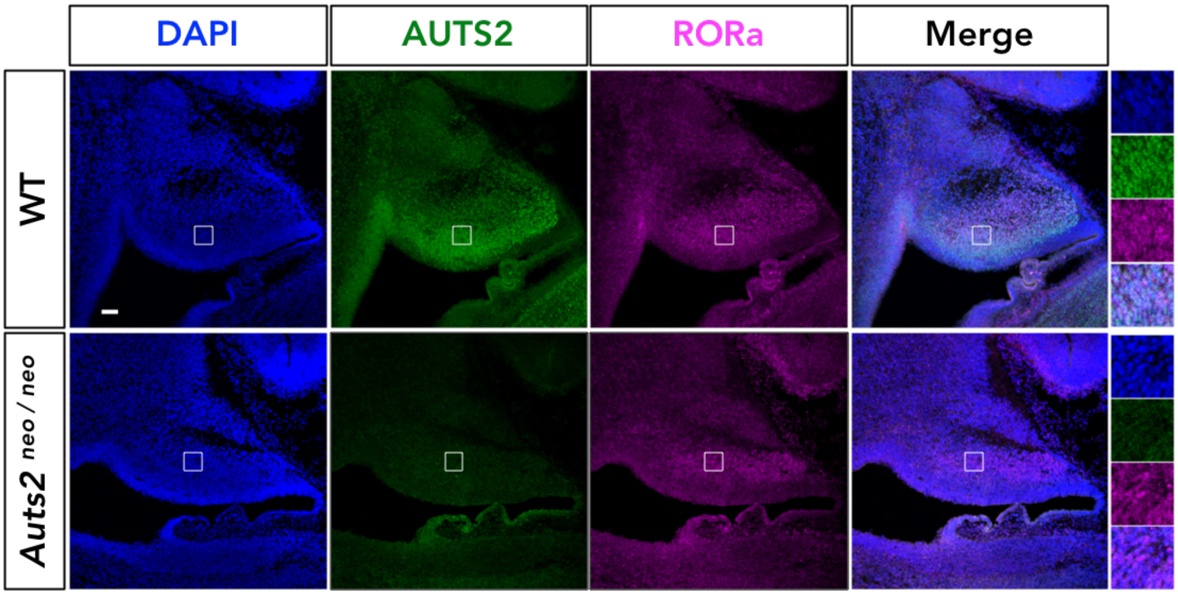

Figure S2. Characterization of the specificity of anti-AUTS2 antibody, Related to Figure 2. Immunostaining on the sagittal sections of the developing cerebellum (E13.5) using the AUTS2 antibody showing the disappearance of AUTS2 immuno-signals in the RORa-positive PCs of *Auts2*$^{neo/neo}$ homozygotic mutants. Scale bar, 50 μm.

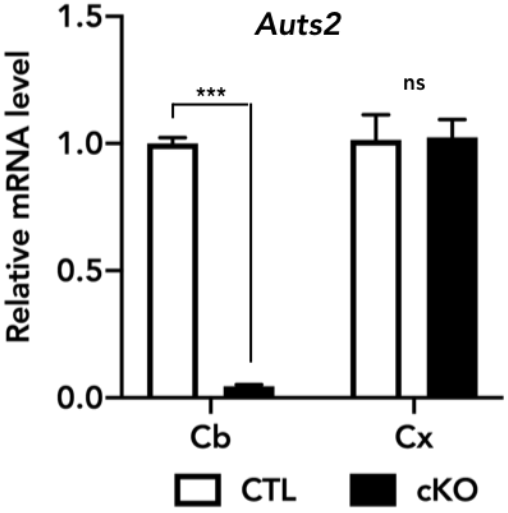

Figure S3. Conditional deletion of *Auts2* gene in *Auts2* cKO cerebellum, Related to Figure 2. *Auts2* mRNA levels in the cerebellum (Cb) and cerebral cortex (Cx) of neonatal *Auts2* cKO homozygotic mutant mice and control mice (CTL). qPCR was performed using primers specific for the deleted exon (n=3-4 mice). Data are shown as mean ± SEM. ***p<0.001, unpaired t-test with Welch's correction.

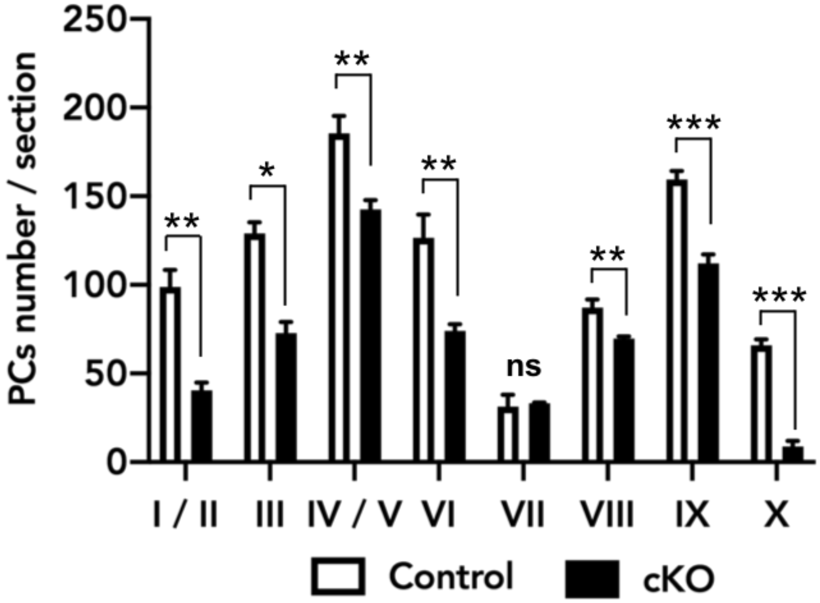

Figure S4. Loss of *Auts2* results in the reduction of PCs, Related to Figure 2.

Graph showing the measurements of the number of PCs at each lobule in sagittal cerebellar vermis sections from adult *Auts2* cKO mutants and control mice (n=4 mice). Data are shown as mean ± SEM. ***$p<0.001$, Mann-Whitney U test.

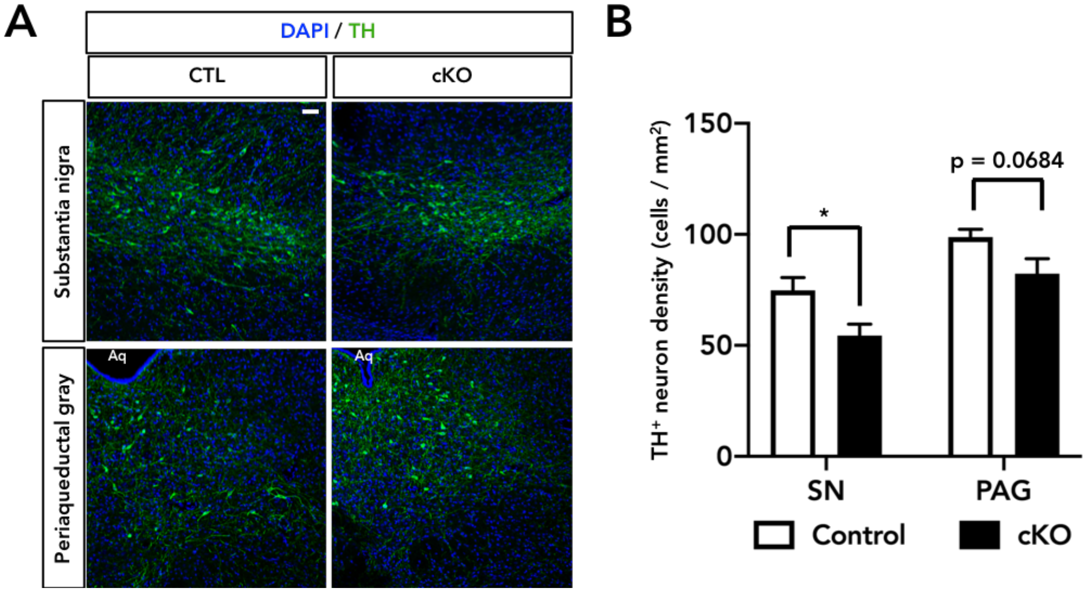

Figure S5. Characterization of the dopaminergic neurons in *Auts2* cKO midbrains, Related to Figure 2.

(A) Immunofluorescence on coronal midbrain sections from P20 control and *Auts2* cKO mice using anti-tyrosine hydroxylase (TH) antibody. Aq: aqueduct of midbrain. (B) Graph shows the density of the TH-positive dopaminergic neurons at substantia nigra (SN) and periaqueductal gray (PAG) in *Auts2* cKO mice and controls (n=4-12 areas, 3 mice). Dopaminergic neurons were reduced in the SN of *Auts2* cKO mutants. Data are presented as mean ± SEM. P<0.05, unpaired t-test. Scale bar, 50 μm.

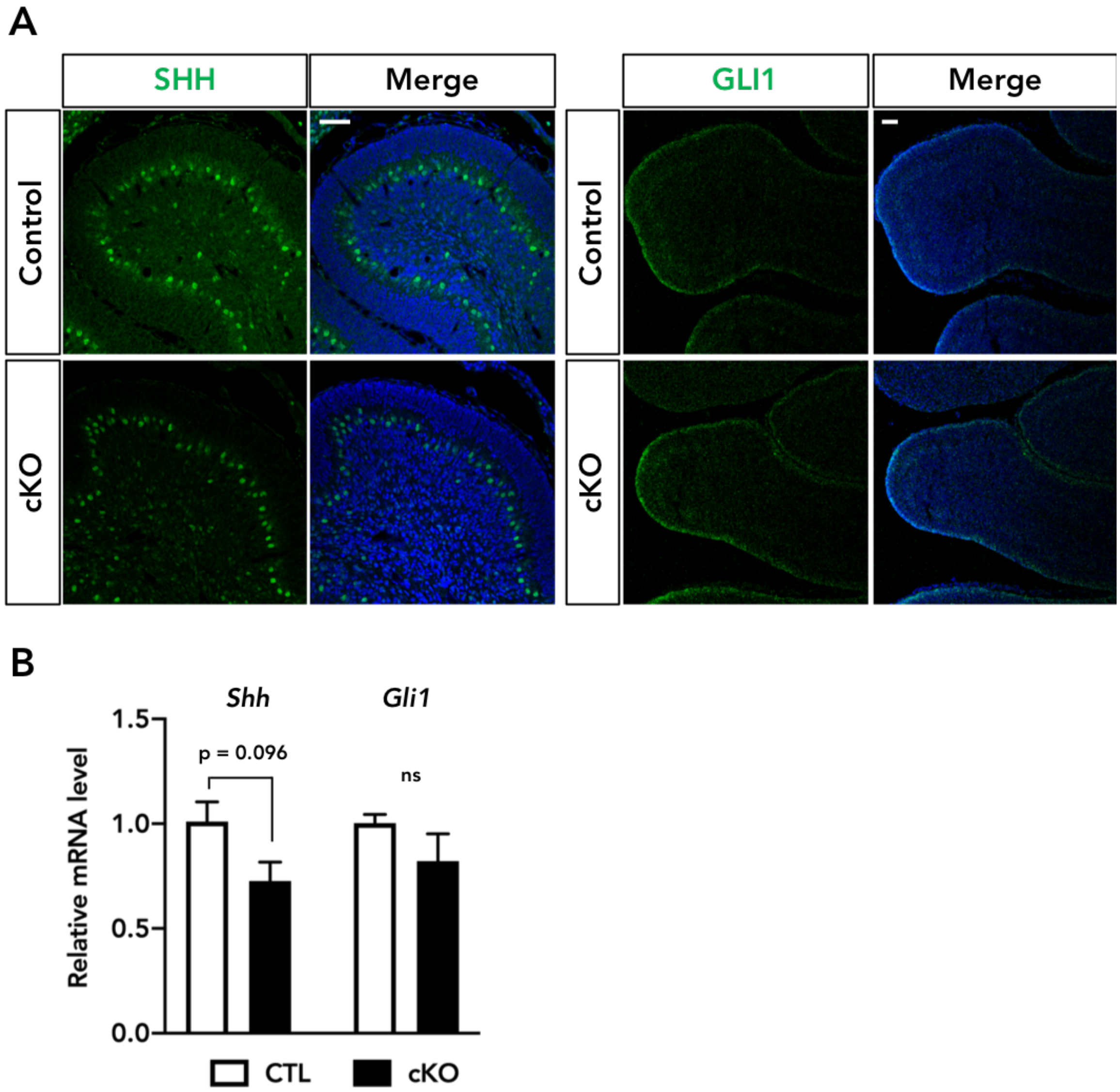

Figure S6. Normal SHH signaling in the developing cerebellum of *Auts2* cKO mice, Related to Figure 2.

(A) Representative images of the postnatal (P7) sagittal cerebellar tissue sections from *Auts2* cKO mutant and control mice stained with SHH (left panels) and GLI1 (right panels). (B) Graph shows the measurements of the transcript levels of SHH-signaling molecules in *Auts2* cKO and control mice (n=3 mice) at P7. Data are shown as mean ± SEM. ns, not significant, unpaired t-test with Welch's correction.

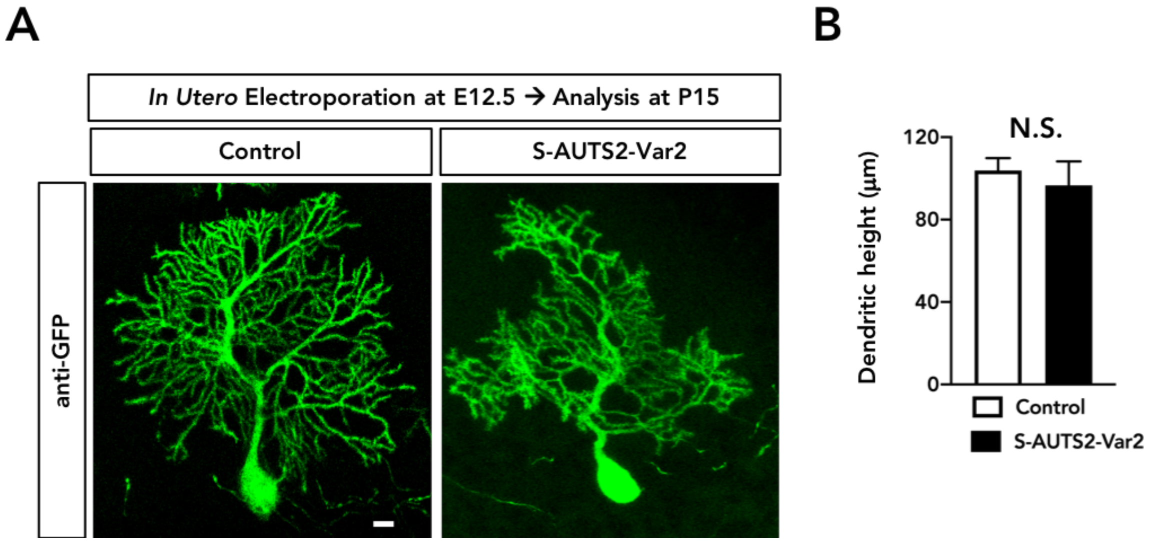

Figure S7. Overexpression of S-AUTS2-Var2 does not affect dendritic outgrowth of PCs, Related to Figure 3.

(A) Representative images of control (EGFP) and S-AUTS2-Var2-overexpressed PCs at P15. The expression vectors were electroporated into WT embryonic cerebellum at E12.5 and tissues were analyzed at P15. (B) Measurement of dendrite lengths of PCs toward the pial surface. n=6-8 cells from N=2-3 mice. N.S., not significant, Mann-Whitney U test. Scale bar, 20 μm.

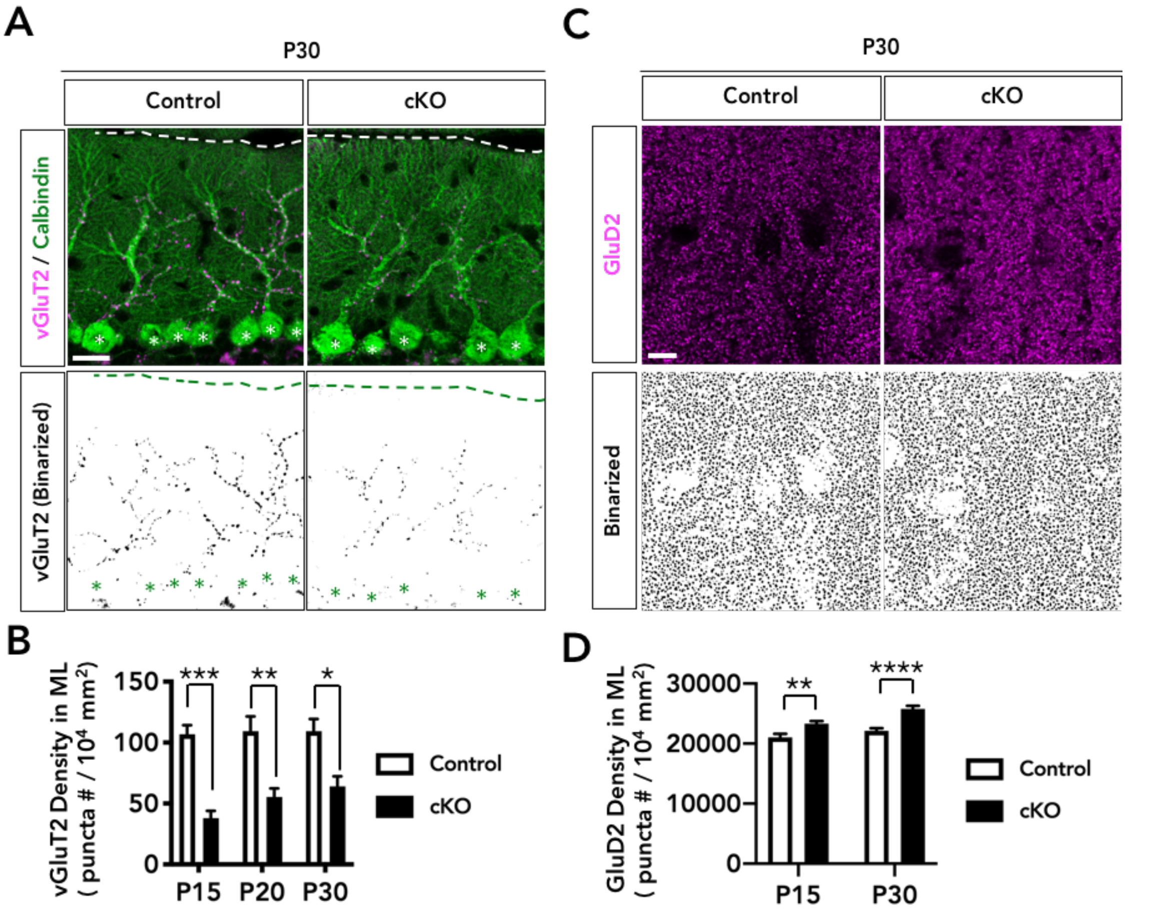

Figure S8. The density of CF and PF synapses in *Auts2* cKO mice during postnatal development, Related to Figure 4.

(A) Representative images showing double immunostaining with Calbindin (green) and vGluT2 (magenta) in P30 control and *Auts2* cKO mice in lobule IV/V. The vGluT2 immunoreactivities are binarized in the bottom. Dotted lines and asterisks indicate pial surface of the ML and PC soma, respectively. (B) Quantification of the density of vGluT2 puncta in ML from P15 to P30 control and *Auts2* cKO cerebellum (n = 4-6 areas from N = 2-3 mice). (C) Representative images showing immunostaining with GluD2 (magenta) in P30 control and *Auts2* cKO mice in the ML with high magnification. The GluD2-immunoreactivities are binarized at the bottom. (D) Measurements of the GluD2 puncta density in ML of control and *Auts2* cKO mice at P15 and P30 (n = 40-54 areas from 3 mice). Data are shown as mean ± SEM. *$p < 0.05$, **$p < 0.01$, ***$p < 0.001$, ****$p < 0.0001$ by unpaired t-test with Welch's correction in (B) and Mann-Whitney U test in (D). Scale bar, 20 μm in (A) and 5 μm in (C).

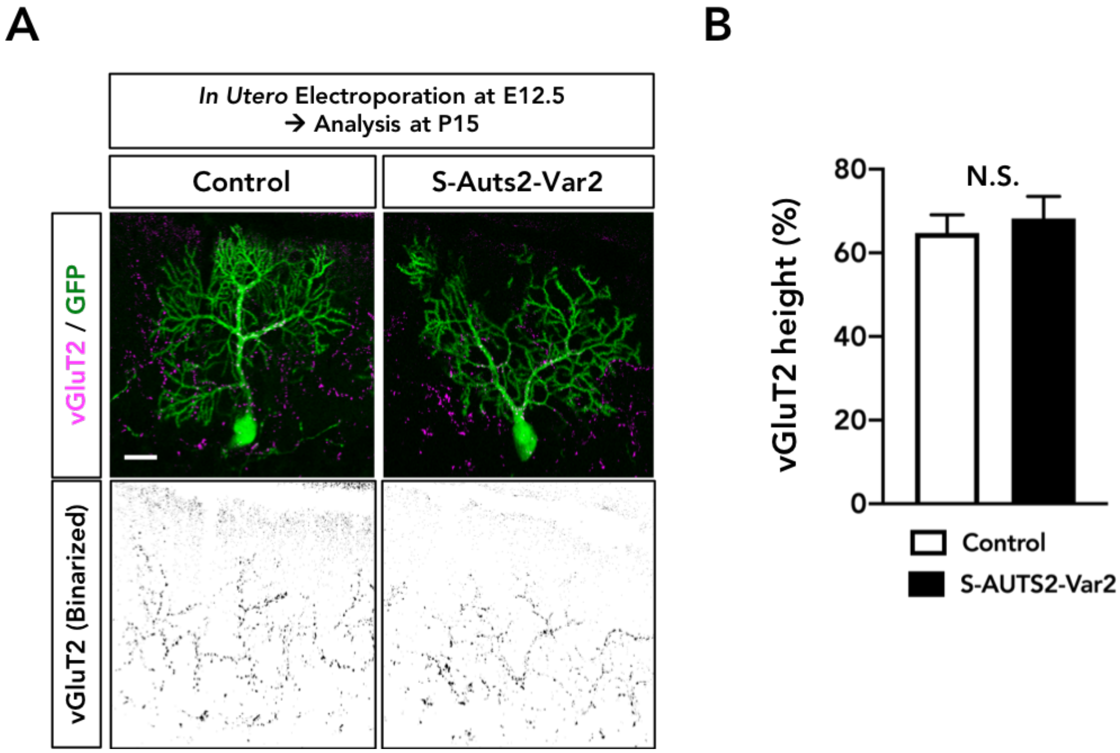

Figure S9. S-AUTS2-Var2-overexpressed PCs exhibit normal CF translocation, Related to Figure 4.

(A) Representative images of EGFP (control) and S-AUTS2-Var2-overexpressed PCs at P15. The expression vectors were introduced by *in utero* electroporation into WT embryonic cerebellum at E12.5 and cerebellar tissues stained with anti-GFP (green) and vGluT2 (magenta) were analyzed at P15. The vGluT2-immunoreactivities are binarized at the bottom. (B) Quantitative analysis of the ratio of vGluT2 height to the tip of PC dendrites (n =6 cells from 3 mice). Data are shown as mean ± SEM. N.S., not significant, unpaired t-test. Scale bar, 20 μm.

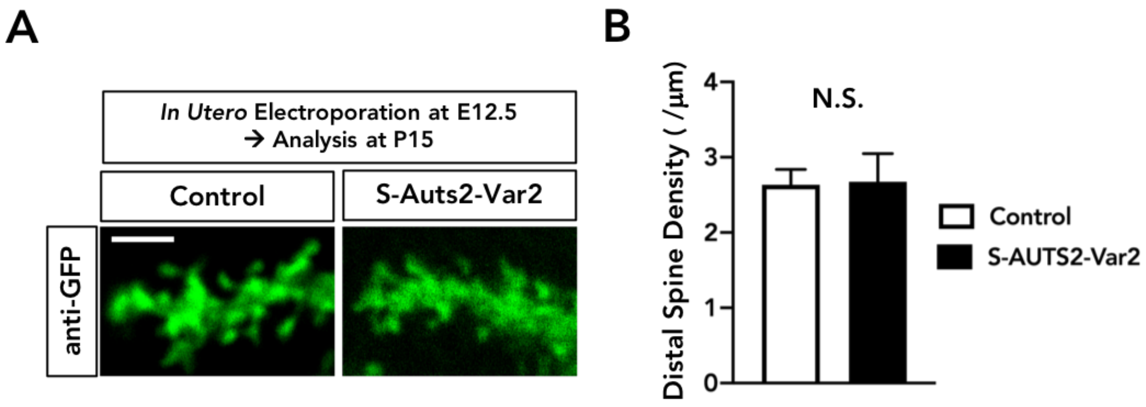

Figure S10. Characterization of dendritic spine formation in S-AUTS2-Var2-overexpressed PCs, Related to Figure 4.

(A) Representative images of the spines on the distal end of dendrites from control (EGFP) and S-AUTS2-Var2-overexpressed PCs at P15. Expression plasmids were electroporated into WT embryonic cerebellum at E12.5. (B) Quantification of the dendritic spine density on the distal end of dendrites from control and S-AUTS2-Var2-overexpressed PCs at P15 (n=5-6 dendrites from 2-3 mice). Data are presented as mean ± SEM. N.S., not significant, Mann-Whitney U test. Scale bar, 2 μm.

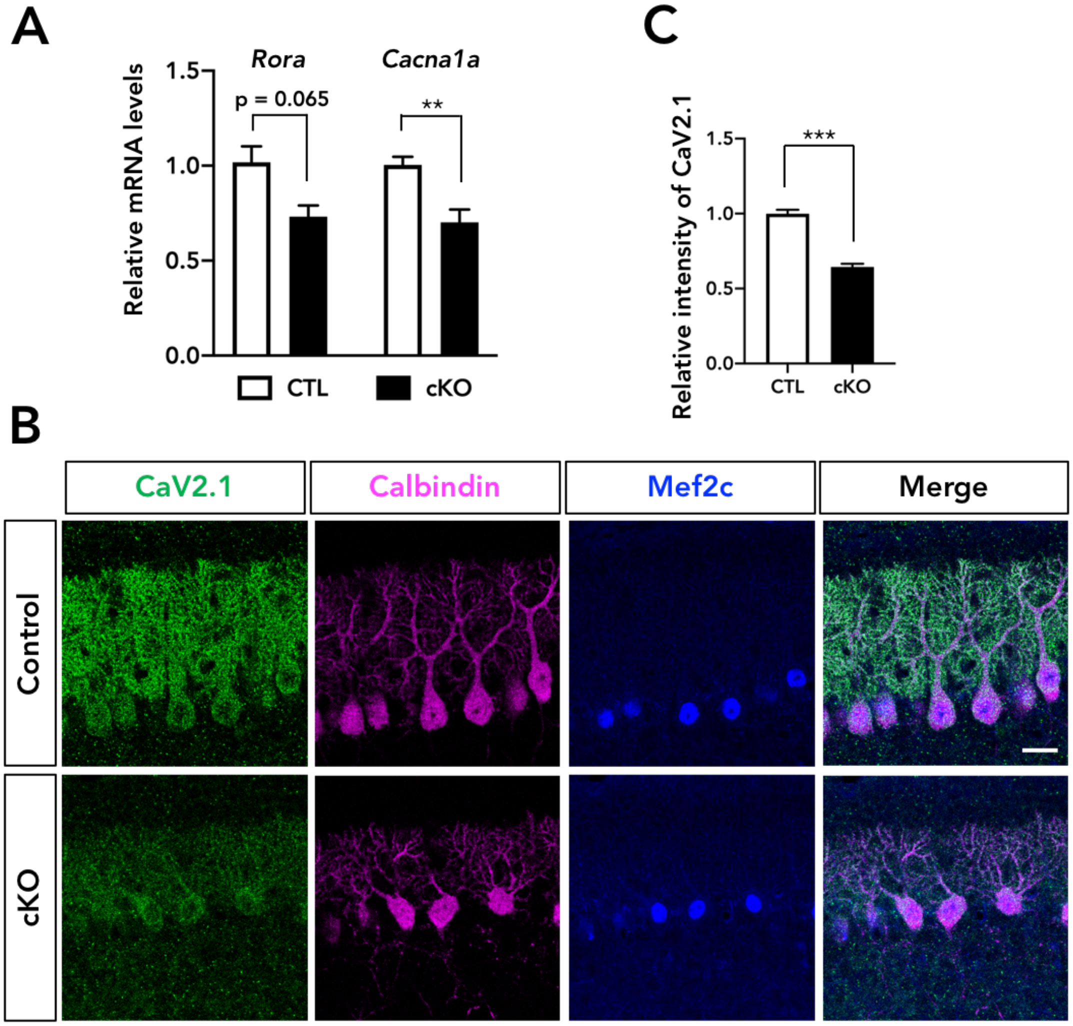

Figure S11. Downregulation of CaV2.1 in the PCs of *Auts2* cKO mice, Related to Figure 4.

(A) qRT-PCR results show that expression levels of *Cacna1a*, but not *Rora*, was significantly reduced in *Auts2* cKO mice at P7 (n = 6 mice). (B) Representative images of triple-immunostaining with CaV2.1(green), Calbindin (magenta) and Mef2c (blue) in P10 control and *Auts2* cKO mice in cerebellar lobule VI. (C) Decreased immunofluorescence intensity levels of CaV2.1 normalized with Mef2c in PCs in control and *Auts2* cKO mice. n=357-375 cells, 3 mice. Data are shown as mean ± SEM. **p < 0.01, ***p < 0.001 by unpaired t-test (A) and Mann-Whitney test (B). Scale bar, 20 μm.

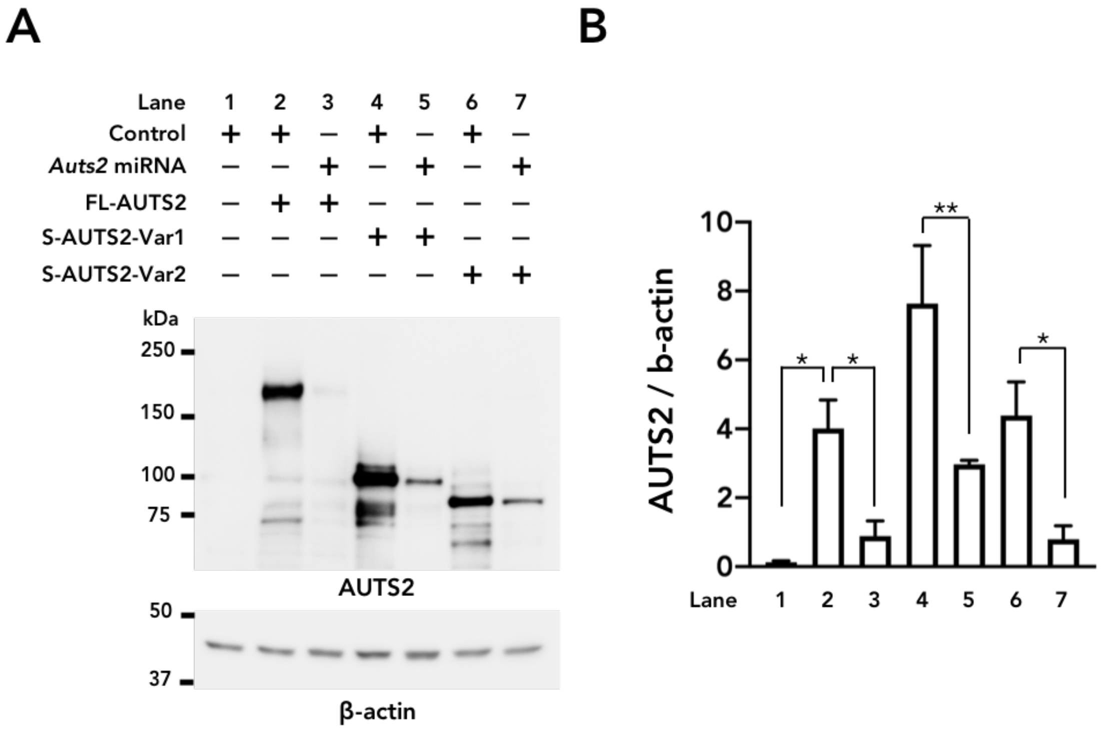

Figure S12. The efficacy of *Auts2* miRNA vector, Related to Figures 5 and 6.

(A) Representative images showing lysates of HEK293T cells transfected with or without control (pCL20c-trL7), *Auts2* miRNA, FL-AUTS2, S-AUTS2-Var1 and S-AUTS2-Var2 expression vector that were immunoblotted with AUTS2 and β-actin antibodies. (B) Quantification of AUTS2 protein levels normalized with β-actin. N = 3 cells for lane 1 (control only), 2 (control and FL-AUTS2), 3 (*Auts2* miRNA and FL-AUTS2), 6 (control and S-AUTS2-Var2) and 7 (*Auts2* miRNA and S-AUTS2-Var2); N = 5 cells for lane 4 (control and S-AUTS2-Var1) and 5 (*Auts2* miRNA and S-AUTS2-Var1). Data are shown as mean ± SEM. *p < 0.05, **p < 0.01, by Mann-Whitney U test or unpaired t-test with Welch's correction in (B).

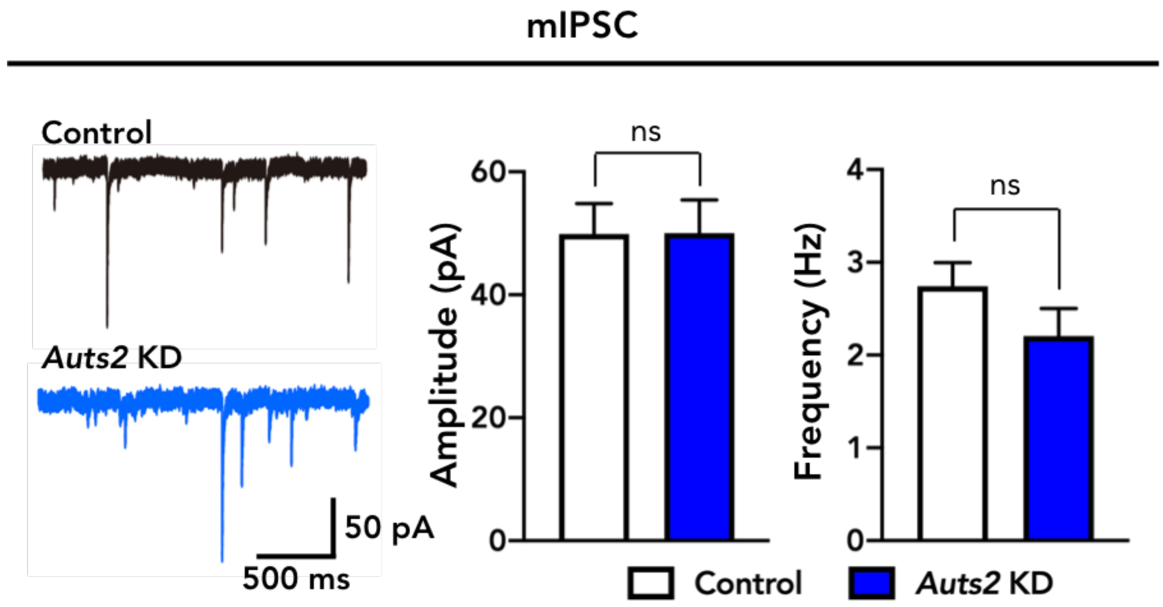

Figure S13. Normal mIPSC in *Auts2*-KD PCs, Related to Figures 5.

No effect of mIPSC in *Auts2*-KD PCs at P20-30. Panels depict representative mIPSC traces (left) and summary graphs of the mIPSC amplitude and frequency in control and *Auts2*-KD PCs. n= 19 cells, 6 mice for control and n= 18 cells, 6 mice for *Auts2* KD. Data are shown as mean ± SEM. ns; not significant, unpaired student t-test.

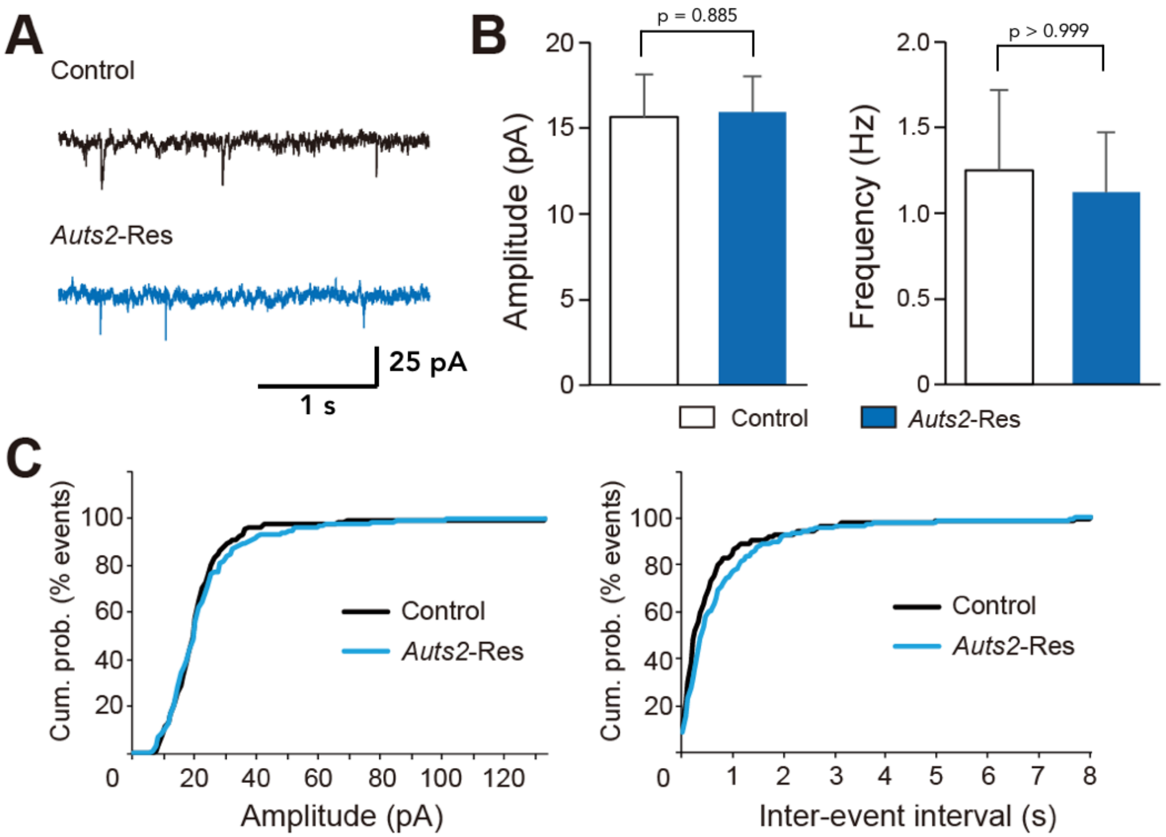

Figure S14. Co-transfection of an RNAi-resistant *Auts2* with the *Auts2* targeted miRNA normalizes enhanced excitatory synaptic transmission by *Auts2* knockdown, Related to Figures 5.

(A) Sample traces of mEPSCs for non-transfected (control) and transfected (*Auts2* KD + *Auts2*-Res) PCs. (B) Summary bar graphs showing the amplitude and frequency of mEPSCs for control (white columns, n = 9 cells from 4 mice) and *Auts2* KD + *Auts2*-Res (blue columns, n = 9 cells from 4 mice) PCs at P20-30. (C) Cumulative probability distributions of mEPSC amplitudes (left) and inter-event intervals (right) in control and *Auts2*-Res PCs. No significant differences were found. Data are shown as mean ± SEM. Mann-Whitney test in (B).

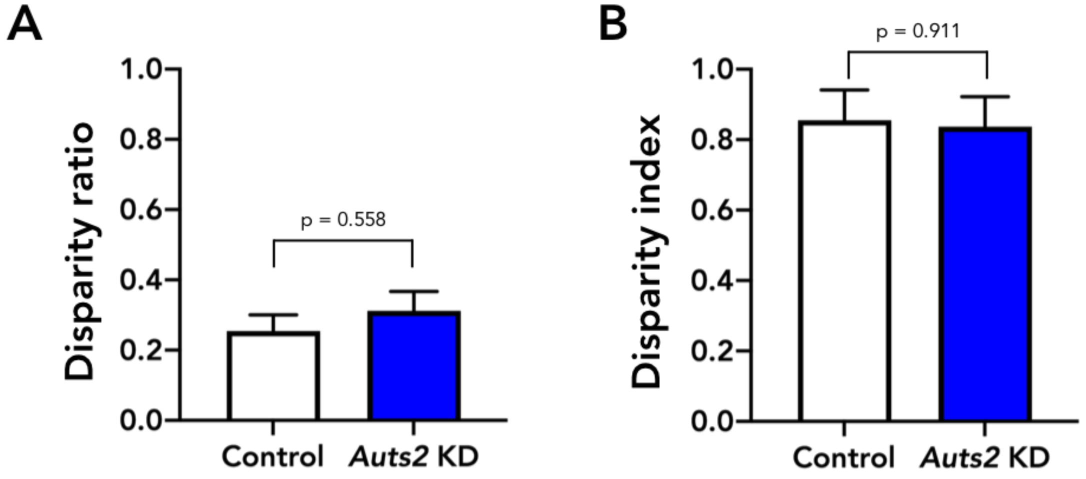

Figure S15. Knockdown of *Auts2* in PCs does not affect the selective strengthening of single CFs at P20-30, Related to Figures 6.

(A, B) Quantification of the disparity ratio (A) showing the relative differences among the strengths of multiple CF-EPSCs, and of the disparity index (B) indicating the coefficient of variation for all CF-EPSC amplitudes measured in a given PC. Methods for calculation are as previously described. n= 6 cells, 3 mice for control and n= 20 cells, 4 mice for *Auts2* KD. Data are shown as mean ± SEM. Unpaired student t-test in (A) and (B).

	Control	*Auts2* KD	P value
Amplitudes (nA)	2.30 ± 0.14	2.93 ± 0.27	0.059
10%-90% Rise Time (ms)	0.47 ± 0.02	0.57 ± 0.03	0.007**
Decay Time constant (ms)	5.26 ± 0.24	4.27 ± 0.24	0.006**
N(cells, mice)	(23, 3)	(26, 4)	

Table S1. Summary of electrophysiological characterization of the strongest CF-EPSCs in control and *Auts2* knockdown PCs at P20-30, Related to Figures 6.

Data are shown as mean ± SEM. p** < 0.01, unpaired Student's t-test.

Transparent Methods

Experimental animals

All animal experiments were conducted in accordance with the guidelines for the Animal Care and Use Committee of the National Center of Neurology and Psychiatry, and for the care and use of laboratory animals of the University of Tokyo and the Japan Neuroscience Society. *Engrailed-1*$^{Cre/+}$ (*En1*$^{Cre/+}$) mice and *Auts2-floxed* mice have been described previously (Hori et al., 2014; Kimmel et al., 2000). *En1*$^{Cre/+}$ mice were obtained from The Jackson Laboratory. *En1*$^{Cre/+}$ mice and *Auts2-floxed* mice were maintained in the C57BL/6N background. *En1*$^{Cre/+}$ *;Auts2*$^{flox/flox}$ homozygous mice were generated by crossing *En1*$^{Cre/+}$ mice with *Auts2*$^{flox/flox}$ mice to obtain *En1*$^{Cre/+}$ *;Auts2*$^{flox/+}$ heterozygous mutant progeny. *En1*$^{Cre/+}$ *;Auts2*$^{flox/+}$ male mice were crossed with *Auts2*$^{flox/flox}$ female mice to yield litters of control mice (*Auts2*$^{flox/+}$ or *Auts2*$^{flox/flox}$), heterozygous (*En1*$^{Cre/+}$ *;Auts2*$^{flox/+}$) and homozygous (*En1*$^{Cre/+}$ *;Auts2*$^{flox/flox}$) mutant mice. Unless otherwise indicated, *Auts2*$^{flox/flox}$ mice were used as the control in this study. Mice were maintained in ventilated racks under a 12-h light/dark cycle. Food and water were provided *ad libitum* in temperature controlled, pathogen-free facilities. Only littermate male mice were used for behavioral tests.

Plasmids

The plasmid construction of pCAG-Myc-AUTS2-full length, pCAG-Myc-AUTS2-var1, pCAG-Myc-AUTS2-var2 were previously described (Hori et al., 2014). For *Auts2* knockdown, the plasmid DNAs were designed to express EGFP and/or microRNA (miRNA) directed against *Auts2* under the control of a truncated L7 promoter (pCL20c-trL7) (Sawada et al., 2010). Engineered microRNAs (5′-TGCTGATAAAGTGGAAGGTCGTGCCAGTTTTGGCCACTGACTGACTGGCACGATTCCACTTTAT-3′ and 5′-CCTGATAAAGTGGAATCGTGCCAGTCAGTCAGTGGCCAAAACTGGCACGACCTTCCACTTTATC-3′) were designed against the mouse *Auts2* coding sequence using the BLOCK-iT Pol II miR RNAi Expression Vector Kit guidelines (Invitrogen). *Auts2* miRNA constructs were subcloned into the pCL20c-trL7. For *Auts2* rescue experiments, the cDNA for *Auts2* was obtained by PCR of a cDNA library from the cerebellum of P10 mice. The QuikChange Lightning site-directed mutagenesis kit (#210518, Agilent Technologies) was used to generate RNAi-resistant forms of *Auts2* (*Auts2*-Res) in which seven nucleotides were mutated without changing the amino acid sequence in the miRNA targeted site. *Auts2*-Res was linked in-frame to EGFP interposed by a picornavirus "self-cleaving" P2A peptide sequence to enable efficient bicistronic expression. The cDNA was subcloned into pCL20c-trL7.

Immunostaining and Nissl staining

Whole brains were dissected out after mice were transcardially perfused with 4% paraformaldehyde (PFA) or periodate-lysine-paraformaldehyde (PLP) in 0.1M sodium phosphate buffer (pH 7.2) under deep isoflurane anesthesia. The brains were further fixed in 4% PFA or PLP for 2 hrs to overnight, cryoprotected with 30% sucrose, embedded in O.C.T. compound (Sakura Fine-Tek, Tokyo, Japan), and cryosectioned at 20 μm. Parasagittal sections were treated with blocking solution containing 1% normal donkey serum (Merck Millipore, Burlington, MA, USA) and 0.2% TX-100 (Nacalai Tesque, Kyoto, Japan) for 1 h at room temperature and immunolabeled using the following primary antibodies in blocking solution at 4 °C overnight: goat-AUTS2 (1:300, EB09003, Everest Biotech, Bicester, UK), rabbit-AUTS2 (1:500, HPA000390, Sigma-Aldrich, St. Louis, MO, USA), rabbit-cleaved Caspase 3 (1:500, 9661S, Cell Signaling Technology, Danvers, MA, USA), rabbit-Calbindin (1:500, AB1778, Merck Millipore), goat-Calbindin (1:1000, Calbindin-Go-Af1040, Frontier Institute, Hokkaido, Japan), rabbit-Neurogranin (1:500, AB5620, Merck Millipore), mouse-Parvalbumin (1:200, P3088, Sigma-Aldrich), guinea pig-vGluT2 (1:500, VGluT2-GP-Af810, Frontier Institute), guinea pig-PSD-95 (1:200, PSD95-GP-Af660, Frontier Institute), rabbit-GluD2 (1:200, GluD2C-Rb-Af1200, Frontier Institute), guinea pig-Car8 (1:200, Car8-GP-Af500, Frontier Institute), Rat-GFP (1:1000, #06083-05, Nacalai Tesque), Rabbit-Mef2c (1:500, D80C1, Cell Signaling Technology), guinea pig-CaV2.1 (1:200, VDCCa1A-GP-Af810, Frontier Institute), rabbit-SHH (1:200, sc-9024, Santa Cruz) and goat-GLI1 (1:500, sc-6153, Santa Cruz). The tissue sections were subsequently labeled with secondary antibodies conjugated with Alexa Fluor 488, Alexa Fluor 568 or Alexa Fluor 647 (1:1000, abcam, Cambrige, UK). Cell nuclei were labeled with DAPI (1:3000, Thermo Fisher Scientific, Waltham, MA, USA) and for immunohistochemistry of AUTS2, antigen retrieval was performed using Target Retrieval Solution (Dako, Carpinteria, CA, USA) in a boiling jar pot for 20 min according to manufacturer's procedure. For immunostaining of PSD-95 and GluD2, the tissue sections were pre-treated with 0.2 mg/ml pepsin in 0.2N HCl for 20 min before primary antibody reaction as previously described with modifications (Fukaya and Watanabe, 2000; Yamasaki et al., 2011). Fluorescent images were acquired with a laser scanning confocal microscope (FV1000, Olympus, Tokyo, Japan) or Zeiss LSM 780 confocal microscope system and ZEN software (Carl Zeiss, Oberkochen, Germany). For Nissl staining, sections were stained with 0.1 % cresyl violet in 1 % acetic acid, dehydrated with ethanol series, mounted in Entellan, and observed with a Keyence All-in-One microscope (BZ-X700, Osaka, Japan). The cerebellar area, the number of PCs, dendritic height and diameter, the height and puncta number of vGluT2 and fluorescence intensities for GluD2-immunosignals were

measured with Fiji software. For measurements of the height and puncta number of vGluT2, puncta with sizes greater than 0.5 μm^2 were defined as vGluT2-positive CF presynapses whereas puncta smaller than 0.5 μm^2 or located outside the dendritic shafts were considered as background noise (Miyazaki et al., 2004). The density of GluD2 puncta was automatically measured and calculated using "Trainable Weka segmentation", "Auto Threshold "and "Analyze Particles" modules in Fiji software with modification.

Immunoblotting

For the detection of endogenous AUTS2 in the cerebellum, whole cerebella were solubilized with SDS sample buffer, and boiled at 95 °C for 5 min. Whole cerebella lysates (1 mg) were fractionated by SDS-PAGE, and transferred onto a nitrocellulose membrane (Bio-Rad, Hercules, California, USA), immunoblotted with primary antibodies including rabbit-AUTS2 (1:500, HPA000390, Sigma-Aldrich, St. Louis, MO, USA), rabbit-GAPDH (1:1000, 2118S, Cell Signaling Technology), and then visualized using HRP-conjugated secondary antibody (GE Healthcare, Chicago, IL, USA) followed by ECL prime (GE Healthcare, Chicago, IL, USA).

For the evaluation of knockdown efficacy, HEK293T cells were simultaneously transfected with or without pCL20c-trL7, *Auts2* miRNA, FL-AUTS2, S-AUTS2-Var1 and Var2 using Lipofectamine LTX Reagents (15338100, Invitrogen) according to manufacturer's instructions. At 36-48 hrs after transfection, cells were harvested, solubilized with SDS sample buffer and boiled at 95 °C for 5 min. Samples were fractionated by SDS-PAGE with NextPage III gradient gels (GLX-3YGM, Gellex, Tokyo, Japan), transferred to nitrocellulose membranes, immunoblotted with primary antibodies including rabbit-AUTS2 (1:500) and mouse-β-Actin (1:1000, 6D1, MBL, Nagoya, Japan) and visualized with HRP-conjugated secondary antibody. Images were acquired by a cooled CCD camera (LAS-4000 mini; Fujifilm, Kanagawa, Japan).

Golgi staining

Whole brains were subjected to Golgi impregnation solution (FD Rapid GolgiStain kit, FD NeuroTechnologies, Columbia MD, USA). Parasagittal sections at 80-100 μm thickness were prepared with cryostat (CM3050S, Leica, Germany) and mounted on gelatin-coated slides. Golgi-Cox staining was performed according to manufacturer's instructions. The sections were dehydrated with an ethanol series and embedded in Entellan (Merck, Darmstadt, Germany). After z-stack images of dendritic spines were captured using 3-zoom mode of Keyence microscope with a 100x oil-immersion objective, the length of dendrite and number of spines were manually quantified

using Fiji software.

Quantitative RT-PCR

Total RNA from whole cerebella at P7 was purified with the Qiagen RNeasy Plus Universal mini kit (Qiagen, Hilden, Germany). One µg of purified RNA was reverse transcribed to cDNA using the ReverTra Ace qPCR RT kit (Toyobo, Osaka, Japan). Real-time qPCR was performed with PowerUp SYBR Green Master Mix (Thermo Fisher Scientific, Waltham, MA, USA) with the Light Cycler® 96 system (Roche, Basel, Switzerland). The relative expression was calculated via the 2Δ method and normalized to β-actin as the internal control. Primer sequences are as follows: *Rora*, fwd 5′-GTGGAGACAAATCGTCAGGAAT-3′ and rev 5′-TGGTCCGATCAATCAAACAGTTC-3′; *Cacna1a*, fwd 5′- CACCGAGTTTGGGAATAACTTCA-3′ and rev 5′- ATTGTGCTCCGTGATTTGGAA-3′; *Actb*, fwd 5′-GGCTGTATTCCCCTCCATCG-3′ and rev 5′- CCAGTTGGTAACAATGCCATGT-3′.

***In utero* electroporation (IUE)**

IUE experiments were performed as previously described (Takeo et al., 2015). In brief, pregnant C57BL/6 mice at E11.5 or E12.5 obtained from CLEA Japan (Tokyo, Japan) and Japan SLC (Tokyo, Japan) were deeply anesthetized with sodium pentobarbital (50-60 µg/g of body weight, intraperitoneal injection). Plasmid DNAs were dissolved in HEPES-buffered saline at a final concentration of 1-2 µg/µl together with Fast Green (0.3 mg/mL). The plasmid solution (1-3µl) was injected into the fourth ventricle by air pressure under the illumination of a flexible fiber optic light source and electrical pulses (33V, with a duration of 30ms, at intervals of 970 ms per pulse, 5 cycles) were applied with tweezer-type electrodes (CUY650P3; NEPA Gene, Chiba, Japan) and an electroporator (CUY21SC; NEPA Gene, Chiba, Japan).

Electrophysiology

The procedures for electrophysiological recordings have been described previously (Hashimoto and Kano, 2003). Briefly, mice at P21-30 were decapitated under CO_2 anesthesia, and brains were rapidly removed and placed in chilled external solution (0-4°C) containing 125 mM NaCl, 2.5 mM KCl, 2 mM $CaCl_2$, 1 mM $MgSO_4$, 1.25 mM NaH_2PO_4, 26 mM $NaHCO_3$, and 20 mM glucose, bubbled with 95% O_2 and 5% CO_2 (pH 7.4). Parasagittal cerebellar slices (250 µm) were prepared by using a vibratome slicer (VT-1200S, Leica, Germany). Whole-cell recordings were made from visually identified or fluorescent protein-positive using upright and fluorescence microscopes at 32°C (BX50W1, Olympus). We randomly chose the PCs in multiple lobules. Patch pipettes (1.5–2.5 MΩ)

were filled with an intracellular solution composed of (in mM): 60 CsCl, 10 D-gluconate, 20 TEA-Cl, 20 BAPTA, 4 $MgCl_2$, 4 ATP, 0.4 GTP, and 30 HEPES (pH 7.3, adjusted with CsOH) for recording EPSCs; 124 CsCl, 10 HEPES, 10 BAPTA, 1 $CaCl_2$, 4.6 $MgCl_2$, 4 ATP, 0.4 GTP (pH 7.3, adjusted with CsOH) for recording miniature IPSCs (mIPSCs). The pipette access resistance was compensated by 70%. Signals were sampled at 0.1-10 kHz and low-pass filtered at 0.05-2 kHz using an EPC10 patch clamp amplifier (HEKA-Electronik, Lambrecht/Pfalz, Germany). Picrotoxin (100 μM, Nacalai Tesque) and tetrodotoxin (0.5 μM, Nacalai Tesque) were added for recording miniature EPSCs (mEPSCs). NBQX (10 μM, Tocris), R-CPP (5 μM, Tocris) and tetrodotoxin (0.5 μM) were added for recording mIPSCs. Picrotoxin (100 μM) was added to block inhibitory synaptic transmission for recording climbing fiber-induced EPSCs (CF-EPSCs) and parallel fiber-induced EPSCs (PF-EPSCs). Holding potential was -10 mV for CF-EPSCs and -70 mV for PF-EPSCs, mEPSCs and mIPSCs (corrected for liquid junction potential). Stimulation pipettes (5-10 μm tip diameter) were filled with the normal ACSF and used to apply square pulses for focal stimulation (duration of 100 μs, amplitude of 0 V to 100 V). CFs were stimulated in the granule cell layer at positions 20-100 μm away from the PC soma. Single or multiple steps of CF-EPSCs were elicited in a given PC when the intensity of stimulation was increased gradually. The numbers of CFs innervating the recorded PC was estimated based on the number of discrete CF-EPSC steps elicited on that PC (Hashimoto and Kano, 2003). PFs were stimulated in the molecular layer at the position where a maximum response was elicited with the stimulus current of 5 μA. The stimulus intensity was decreased gradually from 5 to 0.5 μA to obtain the input-output curve. Online data acquisition and offline data analysis were performed using PULSE and PULSE FIT software (HEKA-Electronik) or Minianalysis Program ver. 6.0.3 (Synaptosoft Inc, Fort Lee, NJ, USA).

Elevated platform test

Elevated platform tests were carried out as previously described (Alvarez-Saavedra et al., 2014) with modifications. In brief, mice at the ages of 2-4 months were placed in the center of a 10 cm^2 round and 20 cm height elevated platform. The time mice remained on the platform was measured.

Rotarod

Mice at 2-4 months old were placed on an accelerating rod using Rotarod 47600 (Ugo Basile, Italy). After mice were placed on the rod at 4 rpm for 10 sec, the rod was set to accelerate from 4 rpm to 40 rpm over 300 s. Mice were subjected to 3 trials per day for 2 consecutive days with 10 min interval between each trial. The time was measured until they fell from or clung to the rotating drum.

Ultrasonic vocalizations (USVs)

Male mice at 8-15 weeks old were individually housed for a week prior to test time to habituate to the testing environment. Then, an unfamiliar 3 months old C57BL6/N wild type female mouse was placed into the test cage. Recordings started after USVs were detected and continued for 1 min. If USV was not detected for 100 s after male mice met female mice, the number of USVs was regarded as zero. USV recordings were acquired with an UltraSoundGate system (Avisoft bioacoustics, Glienicke, Germany) composed of a CM16/CMPA condenser microphone, Avisoft-UltraSoundGate 116H computer interface, and Avisoft Recorder software with a sampling rate of 400 kHz. The number of and the duration of calls with tone frequencies between 40-170 kHz were automatically measured by MATLAB-based program USVSEG with modification to mouse USVs (Tachibana et al., 2014).

Statistical analysis

Sample size was determined by established methods. Data analyses were performed blinded to the genotype. All statistical analyses were performed by GraphPad Prism 8 (GraphPad Software, La Jolla, CA, USA) and IBM SPSS statistics 21 (IBM SPSS Inc., Chicago, IL, USA). The Shapiro-Wilk test was used for the confirmation of the normal distribution and if significant, a nonparametric Mann Whitney U test was applied for comparison. In case of comparison between two groups, the data within normal distribution and equal variance were analyzed using a two-tailed unpaired t-test. If equal variance of the data was significantly different, we used a two-tailed unpaired t-test with Welch's correction. In case of comparison of more than two groups, two-way ANOVA followed by Bonferroni's multiple comparisons test.

Supplemental References

Alvarez-Saavedra, M., De Repentigny, Y., Lagali, P.S., Raghu Ram, E.V., Yan, K., Hashem, E., Ivanochko, D., Huh, M.S., Yang, D., Mears, A.J., *et al.* (2014). Snf2h-mediated chromatin organization and histone H1 dynamics govern cerebellar morphogenesis and neural maturation. Nat Commun *5*, 4181.

Fukaya, M., and Watanabe, M. (2000). Improved immunohistochemical detection of postsynaptically located PSD-95/SAP90 protein family by protease section pretreatment: a study in the adult mouse brain. J Comp Neurol *426*, 572-586.

Sawada, Y., Kajiwara, G., Iizuka, A., Takayama, K., Shuvaev, A.N., Koyama, C., and Hirai, H. (2010).

High transgene expression by lentiviral vectors causes maldevelopment of Purkinje cells in vivo. Cerebellum *9*, 291-302.

Tachibana, R.O., Oosugi, N., and Okanoya, K. (2014). Semi-automatic classification of birdsong elements using a linear support vector machine. PLoS One *9*, e92584.
